# 4-Aminoquinoline as a privileged scaffold for the design of leishmanicidal agents: structure–property relationships and key biological targets

**DOI:** 10.3389/fchem.2024.1527946

**Published:** 2025-01-29

**Authors:** Angel H. Romero, Francisco Delgado

**Affiliations:** Grupo de Química Orgánica Medicinal, Facultad de Ciencias, Universidad de la República, Montevideo, Uruguay

**Keywords:** 4-aminoquinoline, *Leishmania*, mitochondria, phagolysosome, immunostimulation

## Abstract

Leishmaniasis is one of the most important neglected tropical diseases, with more than two million new cases annually. It is endemic in several regions worldwide, representing a public health problem for more than 88 countries, in particular in the tropical and subtropical regions of developing countries. At the moment, there are neither approved vaccines nor effective drugs for the treatment of human leishmaniasis for any of its three typical clinical manifestations, and, importantly, the drugs of clinical use have several side effects, require complex administration regimens, present high cost, and are ineffective in many populations due to pathogen resistance. Moreover, beyond the pharmacological exigencies, there are other challenges concerning its parasitic nature, such as its great genetic plasticity and adaptability, enabling it to activate a battery of genes to develop resistance quickly. All these aspects demand the identification and development of new, safe, and effective chemical systems, which must not only be focused on medicinal chemistry and pharmacological aspects but also consider key aspects relative to parasite survival.

In this sense, the quinolines and, in particular, 4-aminoquinoline, represent a privileged scaffold for the design of potential leishmanicidal candidates due not only to their versatility to generate highly active and selective compounds but also to their correlation with well-defined biological targets. These facts make it possible to generate safe leishmanicidal agents targeted at key aspects of parasite survival.

The current review summarizes the most current examples of leishmanicidal agents based on 4-aminoquinolines focusing the analysis on two essential aspects: (i) structure–property relationship to identify the key pharmacophores and (ii) mode of action focused on key targets in parasite survival (*e.g.*, depolarization of potential mitochondrial, accumulation into macrophage lysosome, and immunostimulation of host cells). With that information, we seek to give useful guidelines for interested researchers to face the drug discovery and development process for selective and potent leishmanicidal agents based on 4-aminoquinolines.

## 1 Introduction

Leishmaniasis is one of the most important neglected tropical diseases (NTDs). It is caused by obligate intracellular parasites of *Leishmania* spp. That parasite is transmitted to humans by the bite of dipteran sandflies (Hide et al., 2007; Alvar et al., 2011; Alvar and Arana, 2018; Ryan and Ray, 2004; Torres-Guerrero et al., 2017). The parasite resides within the macrophage, where it differentiates and proliferates. There are approximately 20 species of *Leishmania* parasites that can infect humans, promoting three types of clinical manifestations of the disease: (i) mucocutaneous leishmaniasis (ML), cutaneous leishmaniasis (CL), and visceral leishmaniasis (VL). Each one of them is caused by a specific species of *Leishmania*: *Leishmania infantum* and *Leishmania donovani* promote VL, *Leishmania braziliensis* and *Leishmania mexicana* cause CL in the Americas, and *Leishmania major* causes CL in the Old world), and *L. braziliensis* and *Leishmania amazonensis* are responsible for MC (Hide et al., 2007; Torres-Guerrero et al., 2017). CL is described as lesions in the skin, whereas MC consists of deep lesions with deformation of the mucosa zone as a consequence of a high level of infection (Torres-Guerrero et al., 2017). Meanwhile, the VL is characterized by an over-inflammation or increase of the volume of such organs, including the bazo, the spleen (known as splenomegaly), the liver (hepatomegaly), and lymph nodes (lymphadenopathy) (Hide et al., 2007; Torres-Guerrero et al., 2017). In particular, this last clinical manifestation VL is fatal in more than 95% of untreated cases.

From a demographic point of view, leishmaniasis is prevalent in 98 countries of tropical and subtropical regions, registering between 0.7 and 1.3 million new leishmaniasis cases and between 26,000 and 65,000 deaths annually (World Health Organisation, 2024). The majority of VL cases occur in eight countries: Brazil, Eritrea, Ethiopia, India, Kenya, Somalia, South Sudan, and Sudan; the cases of CL are predominant in Afghanistan, Algeria, Brazil, Colombia, Iraq, Pakistan, and Syria; whereas the cases of MCL are found in South America (Pan American Health Organization, 2023).

**FIGURE 1:**
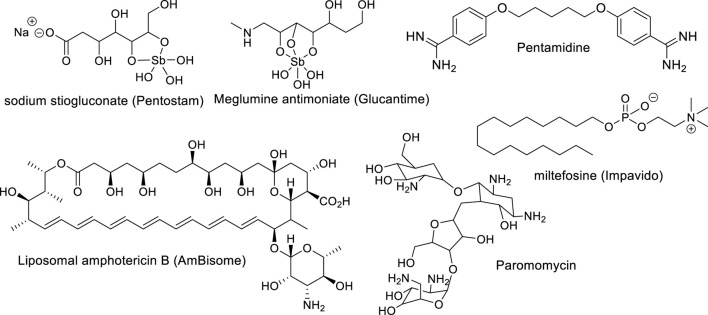
Leishmanicidal drugs approved for clinical use.

Treatment of leishmaniasis represents another challenge for medicinal chemists as a consequence of the high adaptability and genetic plasticity of *Leishmania*. The parasite is able to elude the defense mechanism of macrophage-inducing changes in macrophage polarization as well as to quickly develop resistance to known drugs. Currently, there are neither vaccines nor effective drugs for leishmaniasis treatment in any of its clinical manifestations. There are a few approved drugs, including glucantime, pentostam, pentamidine, amphotericin B, and miltefosine (Figure 1), but they present multiple disadvantages including diverse toxicity manifestations (affecting heart, liver, and kidneys), high cost, low therapeutic efficacy, prolonged administration treatment times (30–60 days) and parasite resistance (Zulfiqar et al., 2017; Aronson et al., 2017). The use of pentavalent antimonials is currently compromised due to the many resistant cases registered in endemic areas of India and Brazil (Aronson et al., 2017). Alternatively, miltefosine has been used for the treatment of VL and CL as the first oral antileishmanial drug; however, its use is also limited by its high toxicity and effectiveness against different *Leishmania* species (Aronson et al., 2017).

**FIGURE 2:**
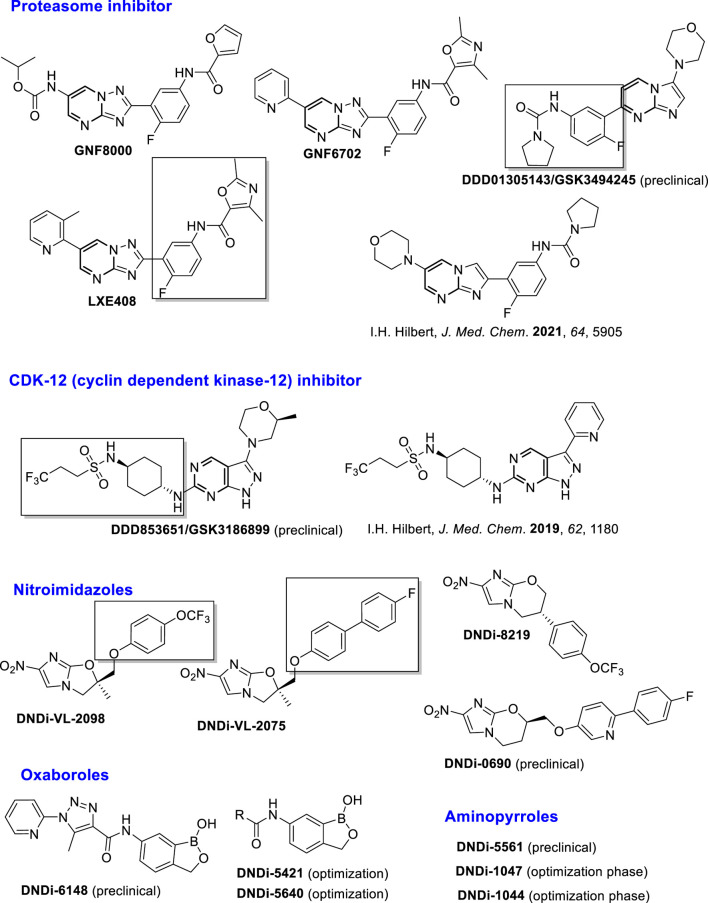
Structure of leishmanicidal leads in the preclinical and clinical phases.

To minimize side-effects, enhance the therapeutic effect, and reduce the emergence of parasite resistance, binary combinations between two of these approved drugs have been employed in specific regions with epidemic situations in Africa and Asia; however, the use of the combination did not provide a definitive solution. This situation has motivated the scientific community to generate new chemical structural alternatives. Many studies have been done by the academy and non-governmental organizations such as Disease Neglected Innovative (DNDi) and Asian and European consortiums. The DNDi presents a portfolio exhibiting a group of compounds that are currently under investigation in preclinical or clinical stages, with a promising therapeutic profile, including (i) proteasome inhibitors such as GNF8000, GNF6702, LXE408, and DDD01305143/GSK3494245 (preclinical stage); (ii) inhibitors of cyclin-dependent kinase-12 (CDK-12), such as DDD853651/GSK3186899 (preclinical stage); (iii) nitroimidazole compounds such as DNDi-VL2098, DNDi-VL2075, DNDi-VL8219, and DNDi-VL0690 (preclinical stage), (iv) benzoxaboroles, such as DNDi-VL6148 (clinical stage) and DNDi-5421 and DNDi-5640 (preclinical stage), and (v) amino-pyrroles such as DNDi-5561, DNDi-1047, and DNDi-1044 (preclinical stage) (Drugs for Neglected Diseases initiative, 2023). Other heterocyclic derivatives developed by Hilbert´s group are in the preclinical phase (Thomas et al., 2019; Thomas et al., 2021) (Figure 2).

Despite the advances, the development of new leishmanicidal drugs remains a significant challenge for medicinal chemists, not only because of the rigorous pharmacological requirements, which seek to minimize side-effect risks, but also because of the genetic plasticity of the parasite and the challenge of effectively accessing a parasite resident in the host cell. That situation calls for the development of new strategies to identify new effective, selective, and safe leishmanicidal agents, which will not only be focused on classic medicinal chemistry concepts but also on critical aspects of parasite survival (Alvar and Arana, 2018). In this sense, quinoline represents a prominent scaffold for the leishmanicidal drug design due not only to its structural versatility for introducing key pharmacophores but also to the plasticity of the chemical system to be targeted on diverse key aspects of parasite life (Silva et al., 2023; Reynolds et al., 2013; Dorababu, 2021). Details concerning the relationship of the quinoline with critical aspects of *Leishmania* life are shown in Section 2.

**FIGURE 3:**
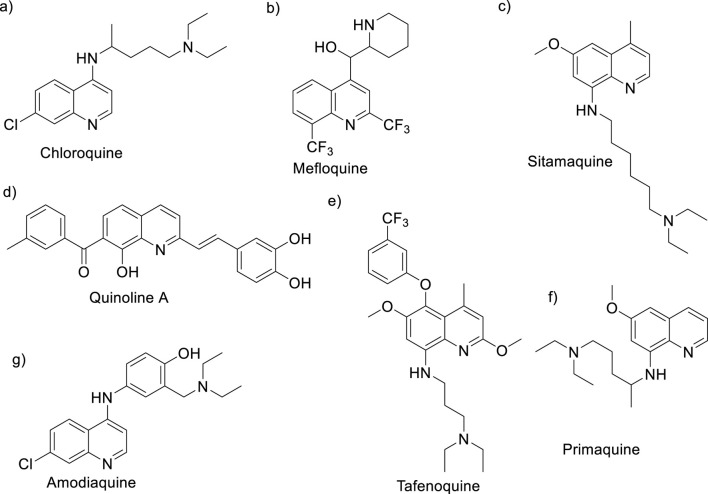
Antimalarial drugs with leishmanicidal activity: **(A)** chloroquine, **(B)** mefloquine, **(C)** sitamaquine, **(D)** quinoline A, **(E)** tafenoquine, **(F)** primaquine, and **(G)** amodiaquine.

When considering quinolines as leishmanicidal agents, there are many examples that feature a variety of pharmacophores (Silva et al., 2023). In the first studies, antimalarial quinolines such as chloroquine, mefloquine, primaquine, sitamaquine, and tafenoquine (Figure 3) were commonly employed upon repurposing program for the discovery of potential quinoline drugs (Reynolds et al., 2013). From active antimalarial agents, sitamaquine (WR 6026, Figure 3) was identified as the most promising quinoline for the treatment of VL not only by its good antileishmanial activity but also its good aqueous solubility for oral administration, excellent pharmacokinetic (short half-time elimination) and ADME parameters (Loiseau et al., 2011; Coimbra et al., 2010). That quinoline drug has reached clinical trials (phase II) for the treatment of VL caused by *Leishmania chagasi* (Dietze et al., 2001); however, its investigation has been stopped due to their adverse effects including methemoglobinemia and nephrotoxicity (Dietze et al., 2001). It is important that the potential of sitamaquine as a leishmanicidal agent is restricted to the treatment of VL because poor or discrete results have been found against *in vivo* models of CL under different topical formulations (Garnier et al., 2006).

Among other antimalarial quinolines used against the *Leishmania* parasite, a group of 8-substituted quinoline such as the 8-hydroxyquinoline A, tafenoquine and primaquine (see structures in Figure 3) have been identified by their excellent leishmanicidal response against *in vitro* infective models (Costa Rocha et al., 2013). The 8-hydroxyquinoline A that presents a better leishmanicidal profile than the reference drug miltefosine is in the preclinical stage within the Drugs for Neglected Diseases initiative (DNDi) program (Loiseau et al., 2011). Tafenoquine exhibits a good leishmanicial *in vitro* profile against a variety of *Leishmania* species as well as a curative response for the *in vivo* murine model of *L. donovani*/BALB/c with a low ED_50_ dose (1.2–3.5 mg/kg for 5 days) (Manzano et al., 2011). Mechanistic studies revealed that tafenoquine (i) targets respiratory complex III with apoptosis consequences and (ii) is able to increase glycolytic ATP synthesis via a sterol-dependent diffusion process. Meanwhile, good *in vitro* and *in vivo* responses for LV models with nontoxic effects were found for primaquine (Shah et al., 2022). However, the potential of the 8-amino/hydroxyquinolines seems to be limited by their tentative side effects (Costa Rocha et al., 2013), making the development of more effective and safer quinolines essential.

In this context, the 4-aminoquinoline emerges as a convenient scaffold, for which a great variety of active compounds against different *in vitro* and *in vivo* models of either VL or CL have been identified (see Section 3). Examples of 4-aminoquinolines include chloroquine, hydroxychloroquine, and an analog such as mefloquine (see structures in Figure 3). Chloroquine has shown a better response against *in vitro* and *in vivo* models of CL than models of VL (Costa Rocha et al., 2013; Hanif et al., 2016; Mwololo et al., 2015). Against intracellular amastigotes of *L. amazonensis* in infected macrophages, for example, the chloroquine and other analogs such as mefloquine and hydroxychloroquine exhibited significant antiamastigote response, with IC_50_ values of 0.78 μM, 1.56 μM, and 0.67 μM, respectively. Interestingly, a more discrete response was found against promastigotes with IC_50_ values higher than 8 µM (Costa Rocha et al., 2013). Studies at the clinical level have shown the curative properties of chloroquine in infected patients, finding better results upon intralesional administration than upon oral administration (Hanif et al., 2016). Meanwhile, amodiaquine, a traditional 4-aminoquinoline antimalarial drug, has also been demonstrated to possess a remarkable antiproliferative effect against different *Leishmania* species with low IC_50_ responses (De Mello et al., 2004). As a consequence of the potential of the 4-aminoquinoline, many active and selective leishmanicidal agents based on 4-aminoquinoline have proved in the last 2 decades against *in vitro* and *in vivo* models of CL and VL, making it possible to recognize specific structural requirements and well-defined mechanistic targets for a rational drug design.

This review provides a general perspective on the key issues to consider for the design of safe and selective leishmanicidal agents based on 4-aminoquinolines. The review analyzed the evolution and development of 4-aminoquinolines as leishmanicidal agents focused on two critical aspects: (i) a recompilation of the typical molecular or cellular targets involving 4-aminoquinoline and (ii) a structure–property relationship analysis among key examples. The structure–property relationship seeks to identify the key pharmacophore from biological potency, selectivity, and physicochemical convenience, whereas the mechanistic insight seeks to identify the key targets for the design of a quinoline with a specific biological response. With that information, we seek to offer readers a tool to face the drug design of leishmanicidal agents based on 4-aminoquinolines.

## 2 Key target insights

This section presents the main biological targets in which the 4-aminoquinolines are involved as leishmanicidal agents. These therapeutic targets are keys to promoting a specific and selective leishmanicidal response, and they have been identified from the *in vitro* and *in vivo* experiments of CL and VL models. Among the most typical targets can be mentioned: (i) accumulation into the mitochondria of the *Leishmania* parasite leading to depolarization of the mitochondrial membrane potential; (ii) accumulation into the macrophage phagolysosome to favor parasite–drug interaction and (iii) immunostimulation of the host cell.

### 2.1 Parasite mitochondria and depolarization of mitochondrial membrane potential

Before describing how 4-aminoquinoline alters the normal mitochondrial potential in the *Leishmania* parasite, it is important to describe some key concepts, including a description of the function of the mitochondria in the *Leishmania* parasite and the role of the mitochondrial potential, its depolarization, and consequences for *Leishmania* life. Mitochondria, the powerhouses of the cell, are organelles found in most eukaryotic cells. Their main function is the cellular respiration and the production of adenosine triphosphate (ATP) through the enzymatic catalysis reaction between the adenosine diphosphate (ADP) and phosphate ion using mitochondrial ATP synthase (Siekevitz, 1957; McBride et al., 2006; Lackner, 2014). Among other tasks, mitochondria are involved in numerous essential functions such as the production of NADH and GTP in the citric acid cycle, the biosynthesis of amino acids, heme groups, and iron-sulfur clusters or the synthesis of phospholipids for membrane biogenesis, calcium signaling, stress responses, and as cellular signaling hubs (Schmidt et al., 2010; Smith et al., 2011; Liew et al., 2021).

**FIGURE 4:**
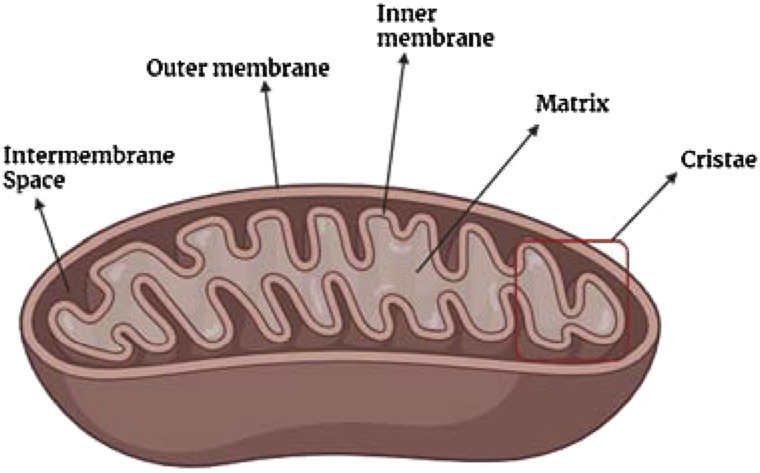
General structure of the mitochondria.

Structurally, a mitochondrion consists of a double membrane structure: the outer membrane and the inner membrane, as depicted in Figure 4. Between the inner and outer membranes of a mitochondrion are three sub-structures: intermembrane space, mitochondrial matrix, and the cristae compartment (Lackner, 2014; Mannella, 2006; Chipuk et al., 2006). The outer membrane is porous, which allows the entrance of ions and small/uncharged molecules through a voltage-dependent ion channel (Lackner, 2014; Mannella, 2006; Chipuk et al., 2006). Other larger molecules, like proteins, tend to enter by special translocases. Meanwhile, the inner membrane is a tight diffusion barrier for diverse types of ions and molecules where oxidative phosphorylation occurs. The mitochondrial matrix, with a pH of 7.9–8 (Lackner, 2014), is the place where the transmembrane electrochemical proton gradient is generated. Also, other processes such as DNA replication, transcription, protein biosynthesis, and numerous enzymatic reactions occur in the mitochondrial matrix (Lackner, 2014). Meanwhile, the intermembrane space, with a pH value of 7.2–7.4, allows the diffusion of chemical components between membranes, whereas the cristae are the location of ATP synthesis (Chipuk et al., 2006).

The *Leishmania* parasite and other trypanosomatids have a single mitochondrion composed of a compact matrix and inner and outer membranes with many cristae (Jensen and Englund, 2012). The size and functions of the *Leishmania* mitochondria are substantially different from those size and functions found in higher eukaryotes (Jensen and Englund, 2012; Souza et al., 2009; Braly et al., 1974; Jardim et al., 2018; Coelho et al., 2010). In particular, the *Leishmania* mitochondria play a role essential in energy production, which is connected with other vital processes in parasite life, such as cellular homeostasis and signaling, biosynthesis of protein and lipid biosynthesis, and beta-oxidation of fatty acids (Jensen and Englund, 2012; Souza et al., 2009; Braly et al., 1974; Jardim et al., 2018). Furthermore, dysfunction of the *Leishmania* mitochondria is correlated with the production of reactive oxygen species (ROS) as well as the promotion of essential cell processes like apoptosis (Ortiz et al., 2016; Menna-Barreto and de Castro, 2014; Opperdoes and Coombs, 2007; Hart and Coombs, 1982). For example, it is reported that an increase in the levels of ROS and lipid peroxide can depolarize the mitochondrial membrane potential (*ΔY*_*m*_) (Ljubava et al., 2018). The mitochondrial membrane potential (*ΔΨ*_*m*_) is a factor derived from redox transformations that occurred during the Krebs cycle, and it serves as a form of energy storage for ATP synthesis (Mitchell, 1966; Glagolev and Skulachev, 1978; Ataullakhanov and Vitvitsky, 2002). Then, the *ΔΨ*_*m*_ in combination with the proton gradient (*ΔpH*) (Mitchell, 1966) generates the transmembrane potential of hydrogen ions that is essential for the ATP synthesis (Glagolev and Skulachev, 1978; Ataullakhanov and Vitvitsky, 2002).

Cell stability and normal cell functioning depend on stable levels of *ΔΨ*_*m*_ and ATP in the cell. A prolonged dysfunction of these key parameters affects the mitochondria function and, consequently, cell viability, promoting a cascade of cell pathologies such as calcium signaling and other key functions (Glagolev and Skulachev, 1978; Ataullakhanov and Vitvitsky, 2002; Zamzami et al., 1995). For example, at a high *ΔΨ*_*m*_, the mitochondrial respiratory chain promotes a significant production of reactive oxygen species (ROS), which have an exponential dependence (Yaniv et al., 2010; Yaniv et al., 2014; Izyumov et al., 2004). Whereas sustained low values of *ΔΨ*_*m*_ are well-documented to minimize the production of ATP as well as to promote “reductive stress” as a consequence of the low ROS production (Jiang et al., 2020).

**FIGURE 5:**
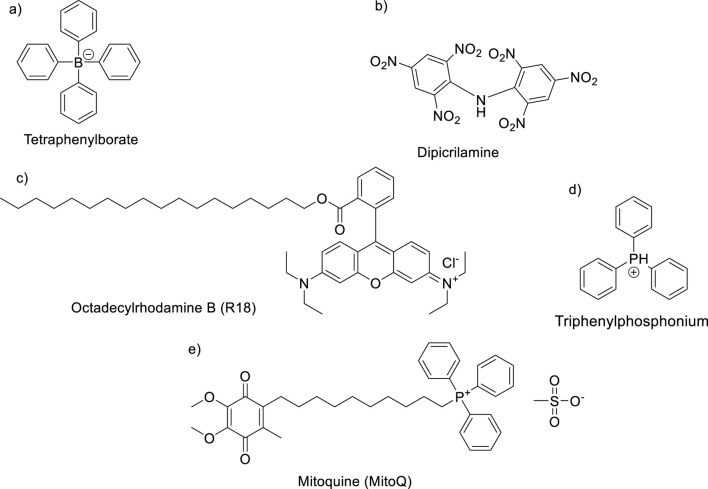
Some typical MTAs: **(A)** tetraphenylborate, **(B)** dipicrylamine (DPA), **(C)** R18, **(D)** TPP, and **(E)** mitoquinone (MitoQ).

*Leishmania* mitochondria represent an attractive target for the drug design, and it is important to understand the rational criteria for a mitochondria-targeted antioxidant (MTAs) (Jiang et al., 2020). The main goal is to seek to alter the normal level of *ΔΨ*_*m*_ using cationic organic compounds taken in advance on the negative value of the mitochondrial membrane potential inside mitochondria and the lipophilic nature of mitochondrial membranes. First, the negative value of the mitochondrial potential favors the accumulation and transportation of permeating cations inside mitochondria (Ross et al., 2005; Zielonka et al., 2017; Robb et al., 2015). The second key aspect is the permeation of the cationic compounds through the mitochondrial membranes. As mentioned earlier, the mitochondrial membranes are composed of lipid bilayer membranes (BLM) and proteins, incorporating lipophilic chains for the design of permeating cationic compounds. The MTAs possess two essential structural features: the existence of a cationic center and the incorporation of large lipophilic chains or lipophilic chemical functions. For example, among the most relevant MTAs can be mentioned tetraphenylborate, dipicrylamine (DPA), octadecyl rhodamine, triphenylphosphonium, and mitoquinone (Ross et al., 2005; Zielonka et al., 2017; Robb et al., 2015; James et al., 2005; Hoye et al., 2008; Blaikie et al., 2006; Biasutto et al., 2010). This type of compound has been shown to cross the BLM and accumulate inside mitochondria (Ketterer et al., 1971; Andersen and Fuchs, 1975; Melikyan et al., 1996; Zimmermann et al., 2008; Liberman and Topaly, 1969) (Figure 5).

The known ligands that target mitochondria are characterized by a lipophilic chain that favors the translocation through the BLM of the cell without a complex uptake mechanism and the presence of a cationic region to accumulate inside the negative mitochondria potential. When discussing *Leishmania*, the miltefosine is the clearest example. It possesses a long lipophilic chain and a cationic region of the terminal ammonium group. More recently, quinolines, including 8-substituted and 4-aminoquinolines, have been correlated with the depolarization of mitochondrial membrane potential into *Leishmania* parasites. In particular, those quinolines with a terminal tertiary moiety and a lipophilic chain in their molecular structure showed a high ability to alter the *ΔΨ*_*m*_ with affectation of cell and mitochondrial functions. For example, sitamaquine is a lipophilic weak base that accumulates in the promastigotes through an electrical gradient involving two steps: i) the interaction between the positively charged sitamaquine with the anionic polar head groups of phospholipids in mitochondrial membranes and (ii) the subsequent insertion of quinoline into the parasite plasma membranes through hydrophobic interaction between the acyl chains of phospholipids and the hydrophobic quinoline chains to enter into the lipid monolayer (Duenas-Romero et al., 2007). It is important to mention that the affinity of sitamaquine by mitochondrial membranes is transitory because it can also be located in the cytosol (Coelho et al., 2010). As a consequence of the internalization in the membrane, the sitamaquine affects the normal electrical potential efflux, generating a rapid collapse of the mitochondrial inner-membrane potential (Lopez-Martin et al., 2008; Vercesi et al., 2000).

**FIGURE 6:**
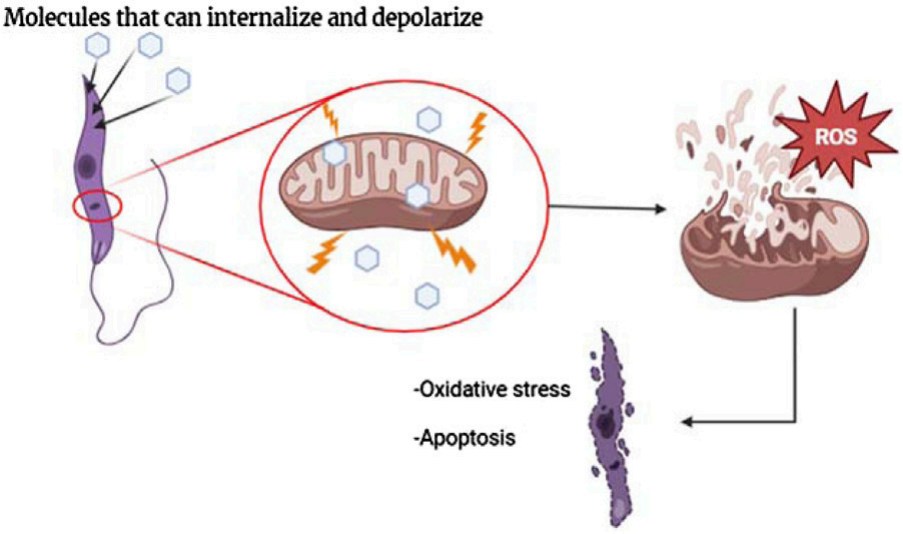
Illustration of the internalization of molecules through the BLM of mitochondria and biological consequences.

This evidence has motivated the design of diverse quinoline compounds, including 4-aminoquinolines targeted toward the *Leishmania* mitochondria. These quinolines are characterized by a weak basic moiety and a lipophilic chain in the quinoline structure, and the leishmanicidal response has shown a consistent correlation with depolarization of mitochondrial potential, promoting a series of biological and biochemical consequences into the parasite, including an increase of ROS production, alteration of parasite morphology and parasite death via apoptosis (Figure 6). In Section 3, we show the effect of a variety of 4-aminoquinolines on the mitochondrial membrane potential of the parasite through the experimental evidence based on the determination of *ΔΨ*_*m*_, level of ROS, alteration of parasite morphology, and evidence of apoptosis. In that section, the influence of this depolarization on the *in vitro* leishmanicidal response is also discussed.

### 2.2 Macrophage lysosome as a target for drug accumulation

The macrophage lysosome is an essential target in the life of the *Leishmania* parasite. As was mentioned earlier, *Leishmania*, an intracellular parasite, lives in the phagolysosomes of mammalian macrophages during its infective stage. The location of the parasite inside the phagolysosome is promoted by the presence of lipophosphoglycan (LPG) along the parasite surface (Desjardins and Descoteaux, 1997; Moradin and Descoteaux, 2012; Séguin and Descoteaux, 2016), which are recognized by parasite facilitating its entrance into phagolysosome. The physiological conditions in the phagolysosome consist typically of an acidic *pH* value of 4–5 and an internal temperature of 37°C. These aspects are critical and play an important role in initiating the phenotypic differentiation of the promastigote form to the amastigote form (Clos et al., 2022). Then, the macrophage lysosome represents an attractive target for the design of novel leishmanicidal drugs, being key to search strategies that favor the accumulation of leishmanicidal drugs into macrophage lysosome. The latter guarantees a direct interaction between the leishmanicidal drug and the parasite, which is key for a direct leishmanicidal effect.

To reach the accumulation of the leishmanicidal drug into the mammalian lysosome (lysosomotropic drug), it is essential to take into account some physicochemical characteristics of the lysosome, such as its internal acidic *pH* and the lipophilic nature of the outer membrane. These chemical characteristics suggest that the lysosomotropic drugs consist of compounds with weak basic groups and a lipophilic chain, which are essential to favor the internal accumulation inside the lysosome via acid–base ionization and the compound penetration through the lipophilic membrane, respectively. A promising study performed by Trapp et al. (2008) based on a comparison of ten known quinoline antimalarial drugs found a high and selective accumulation in lysosomes for weak mono- and bivalent bases with intermediate to high log *K*_*ow*_. The high lysosome accumulation for quinoline bases showed *pK*_*a*_ values between 6 and 10, with an optimum value near 8. The mechanism of this accumulation is the ion trap, involving the uptake of the neutral base into acidic lysosomes with trapping following protonation. The exact maximum concentration ratio of lysosome to outside occurs at *pK*_*a*_ = 7.9. In general, Trapp and co-workers found that the optimum association constant for selective accumulation of bivalent bases in lysosomes is at the 3 < log *K*_*OW*_ < 6 range, whereas for monovalent bases, it is at the 0 < log *K*_*OW*_ < 3 range. For example, chloroquine has a *pK*_*a1*_ value of 9.94 and a *pK*_*a2*_ value of 8.10 (Newton and Kluza, 1978), with a log *K*_*OW*_ of 4.38 (Hansch et al., 1995). Quinacrine exhibited *pK*_*a*_ values of 10.2 and 8.2, with a log *K*_*OW*_ of 4.79 (Hansch et al., 1995). Importantly, the accumulation decreases with an increase in the *pH* of the lysosomal, finding no ion trapping and accumulation when the *pH* in lysosomes reaches the *pH* in the cytosol.

On the other hand, Marceau and co-workers found not only a correlation with the *pK*_*a*_ characteristic but also with the lipophilicity (Marceau et al., 2012). They found an optimum lysosome accumulation for organic bases with *pK*_*a*_ values between 8 and 10, and the accumulation is more effective if the compound presents a Log *P* value between 4 and 6. The findings reported by Trapp and Marceu were validated with experimental results and by comparison to the properties of antimalarial drugs in clinical use, for example, Trapp et al. (2008). Further studies have shown that quinoline rapidly accumulates into acidic compartments like acidocalcisomes (Lopez-Martin et al., 2008). Importantly, an NMR study has demonstrated that sitamaquine does not affect lipid trafficking in *Leishmania* (Coimbra et al., 2010).

**FIGURE 7:**
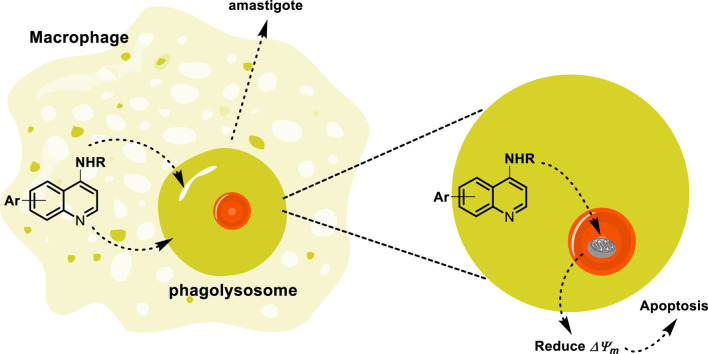
Tentative mechanism of the accumulation of quinoline through the lysosome to reach the mitochondria.

In addition to the lysosome-tropic property of quinoline drugs by mammalian cells, several quinolines have received attention for their antitumor effects, which are favored by their accumulation in the lysosomes of tumor cells. Its accumulation in the lysosome blocks autophagy, prevents the degradation of autophagosomal content, activates the ER stress, and induces apoptosis in tumor cells. The accumulation into lysosome is favored by quinoline protonation under acidic *pH,* decreasing the lysosome function and thus inhibiting autophagy (Solomon and Lee, 2009; McAfee et al., 2012; Degtyarev et al., 2008; Sharma et al., 2012). Recently, the accumulation of quinoline into lysosome in macrophages and mammalian cells has been demonstrated through the use of fluorescence microscopy (Kuo et al., 2016; Fan et al., 2018; Li et al., 2013; Bai and Qian, 2020; Bik et al., 2021). Furthermore, a quinoline compound like Lys05 has demonstrated an effective accumulation into macrophage lysosome via proton trapping, visualized through fluorescence microscopy. Then, the quinoline trapping by parasite phagolysosome not only favors the accumulation into phagolysosome but also facilitates the direct interaction between the quinoline drug and intracellular amastigotes. Although it is still not described, we think that the partial protonation of the basic quinoline into macrophage lysosome generates cationic compounds, which efficiently enter the mitochondria in conjunction with the presence of lipophilic chains, as depicted in Figure 7. Finally, beyond the internalization inside the parasite mitochondria and phagolysosome of leishmanicidal agents based on 4-aminoquinolines, it is reported that sitamaquine can rapidly accumulate into other types of membranous and acidic compartments, mainly into acidocalcisomes (Lopez-Martin et al., 2008; Vercesi et al., 2000). The acidocalcisomes are acidic vacuoles containing most of the cellular calcium, and that type of compartment is present only in trypanosomatids (*Leishmania, Trypanosome cruzi*, and *Trypanosome brucei*) and other flagellated parasites such as *Plasmodium* (causes malaria) and *Toxoplasma gondii* (causes toxoplasmosis), emerging as another convenient target for the drug design of leishmanicidal agents (Docampo et al., 2005).

### 2.3 Host-immunostimulation mediated by toll-like receptor (TLR) agonists

#### 2.3.1 General concepts relative to *Leishmania* infection and the role of TLR

**FIGURE 8:**
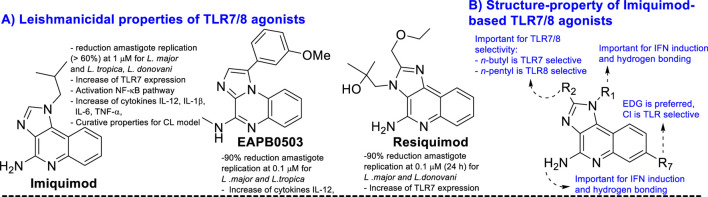
**(A)** Structure of some key TLR agonists of imiquimod, EAPB0503, and resiquimod. **(B)** Structure–property relationship of imiquimod based on TLR 7/8 agonist.

Once the infection within the macrophage is established, the *Leishmania* parasite is able to elude the immune host defense response through the induction of changes in the macrophage polarization from M1 to M2, resulting in a macrophage more focused on differentiation tasks than on defense actions (Tomiotto-Pellissier et al., 2018; Conceicao and Morgado, 2019). Macrophage polarization occurs through the recognition by the macrophage phagocytes of phosphatidylserine that is placed at the surface of apoptotic parasites (Conceicao and Morgado, 2019). This macrophage stage, M2, is characterized by producing low levels of pro-inflammatory cytokines TNF-α, IL-1, IL-6, and IL-12 (Bogdan, 2020; Tomiotto-Pellissier et al., 2018) and high levels of regulatory cytokines like TFG-1β and IL-10. The latter implies a suppression of the lymphocytes and neutrophils and the production of chemical effectors such as nitric oxide and reactive oxygen species (Smirlis et al., 2019). All these features favor the survival of the parasite within the macrophage and provide a path for its colonization toward other macrophages. The use of immunostimulants like imiquimod and resiquimod (Figure 8A) has been demonstrated to control the parasite proliferation for both *in vitro* and *in vivo* infected models. Interestingly, parasite control has shown a good correlation with the increase in the pro-inflammatory cytokine ratio compared with untreated controls (Buates and Matlashewski, 1999; El Hajjldi et al., 2018). These immunostimulants act as agonists of TLR7 and TLR8 receptors. The TLRs are a class of pattern recognition receptors (PRRs), and their signaling pathway represents a primary defense barrier against pathogens to control the activation and progression of adaptive immunity through the production of pro-inflammatory cytokines, interferons, chemokines, and B and T cells (Kawasaki and Kawai, 2014; Tuon et al., 2008; Tuon et al., 2010; He et al., 2013). Macrophages recognize *Leishmania* parasites through TLR2, TLR4, TLR7, TLR8, TLR9, TLR11, and TLR12 (Polari et al., 2019; Vargas-Inchaustegui et al., 2009; Tuon et al., 2010; Tolouei et al., 2013; Raman et al., 2010; Kaushik et al., 2021; Shukla et al., 2018; Faria et al., 2012).

Recent studies have demonstrated a significant parasite proliferation in *Leishmania*-infected Tlr7(^-^1^-^) mice compared to normal infected controls (Faria et al., 2012), which highlights the role of the TLR7 receptor in the immune control against *Leishmania*. Other studies have shown that imiquimod analogs like resiquimod and EAPB0503 (Figures 8A, B) are also able to control parasite proliferation in infected macrophages in combination with stimulation of expression of transcription factors like AP-1 and NF-κB (Smirlis et al., 2019), which are essential for the production of pro-inflammatory cytokines via TLR7 receptor activation. Other types of immunostimulants have controlled parasite proliferation (Regli et al., 2020; Anders et al., 2019; Bernardo et al., 2021; Rodrigues de Santana et al., 2017; Vasilakos and Tomai, 2013; Salyer and David, 2018). It is important to mention that either imiquimod or resiquimod consists of a quinoline structure. In addition, imiquimod and resiquimod are characterized by a weak leishmanicidal response against promastigote and axenic amastigote forms of the parasite, which are the parasite control in the infected model associated with immunostimulant activity (Smirlis et al., 2019; Buates and Matlashewski, 1999). Curiously, most of the proven 4-aminoquinolines have exhibited a significant antiamastigote response against the infected model and a weak leishmanicidal response against axenic forms of the parasite (see Section 3), which suggests a strong association with immunostimulation of the host cell. The ideal situation is the discovery of a leishmanicidal 4-aminoquinoline with a dual leishmanicidal/immunostimulant response. In Section 2.3.2, the use of 4-aminoquinolines as TLR agonists and antagonists is discussed.

#### 2.3.2 4-Aminoquinolines as TLR agonist and antagonist

**FIGURE 9:**
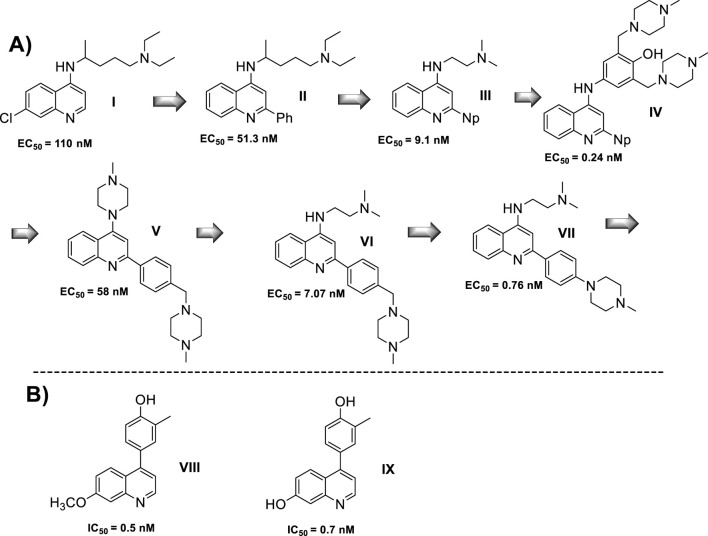
**(A)** Structure progression of 4-quinolines as antagonists of TLR with their corresponding EC_50_ values. **(B)** Derivatives of 4-phenylquinoline as TLR agonists.

A quinoline with a tertiary amine group is a chemical platform involved in immunostimulant response either as a TLR agonist or as a TLR antagonist (Talukdar et al., 2021). In particular, the 2-aminoquinolines have been extensively studied, achieving the discovery of a potent agonist of the TLR8, including some of their analogs such as imiquimod, resiquimod, gardiquimod, which are among the best known (Talukdar et al., 2021). The protonation of the tertiary amino group and the quinolinic nitrogen atom in quinolinic compounds such as chloroquine and quinacrine favors the binding to DNA, emerging as a tool for the inhibition of CpG-DNA (Manzel et al., 1999). Chloroquine was recognized by its CpG-ODN inhibitory effect with an antagonist TLR response. A SAR study of quinoline analogs found that the incorporation of aryl-substituent at the C-2 position, such as phenyl (EC_50_ = 51 nM) or C-2-naphthyl (EC_50_ = 9 nM) enhanced the CpG-ODN inhibitory activity by 2-fold and 12-fold, respectively, compared with chloroquine (EC_50_ = 110 nM) (Figure 9). Interestingly, a correlation between the *pK*_*a*_ and leishmanicidal activity was found, where higher *pK*_*a*_ values generate greater antagonistic response (Strekowski et al., 1999). Then, either the basicity or the lipophilicity are key features for designing greater antagonistic TLR based on 4-aminoquinolines. Further structures showed that an amodiaquine analog with a phenyl linker between 4-amino and terminal tertiary amine compared with chloroquine was significantly more potent as a TLR antagonist (compounds **I-V**, Figure 9A). Its prominent compound displayed an EC_50_ value of 0.24 nM. Subsequently, the naphthyl moiety at the C2 position was replaced by a phenyl-type substituent because the naphthyl moiety is not metabolically stable and is potentially toxic. Other SAR analysis showed that steric terminal dialkylamino at the 4-position compromises the antagonist-receptor interaction, being preferred to a flexible dialkylamino over a cyclic or rigid one like piperazine. Further developments show that compounds **VI** and **VII**, which are chloroquine analogs with a 4-(*N*-methylpiperazine)methyl-phenyl and 4-(*N*-methylpiperazine)phenyl moieties, exhibited a significant antagonist TLR response with EC_50_ values of 7.07 nM and 0.76 nM, respectively (Figure 9A) (Strekowski et al., 2003). Finally, quinoline analogs **VIII** and **IX** that bear the (4-hydroxyphenyl) substituent at the 4-position showed a potent antagonist response against hTLR and gave IC_50_ values of 0.5 nM and 0.7 nM, respectively (Figure 9B) (Zhang et al., 2018; Hu et al., 2018).

In summary, lipophilic quinoline with a strongly basic chain moiety is required for the construction of an antagonist of TLR, which could be of great importance for the immunostimulation of infected macrophages against intracellular amastigotes of *Leishmania*. In Section 3, the role of the immunostimulation of the host cell is discussed and analyzed for a group of 4-aminoquinolines.

## 3 Structure–activity relationship vs. mechanism of action

4-Aminoquinoline is the most versatile quinoline scaffold for the construction of active and potent leishmanicidal agents. In particular, the most promising 4-aminoquinolines are characterized by a tertiary amine or/and a lipophilic group (*e.g.*, alkyl or aryl) into the amino chain. In this section, we performed a structure–activity relationship comparison to provide a general perspective about the key structural features for the design of potent leishmanicidal agents based on 4-aminoquinoline and a direct correlation with the three mentioned targets.

Within the 4-aminoquinolines, chloroquine was one of the first antimalarial quinoline compounds to test against *Leishmania* models, with an excellent *in vitro* response against intracellular amastigotes of the *L. amazonensis* parasite (Costa Rocha et al., 2013). In a clinical trial, chloroquine appeared to be as effective as tetracycline for the treatment of CL (Hanif et al., 2016). Its combination with diminazene provided good leishmanicidal responses that merit further development (De Mello et al., 2004), whereas its combination with paromomycin did not yield encouraging results. In summary, chloroquine displayed a good leishmanicidal response against CL models, but its leishmanicidal response is discrete or weak against VL models (Mwololo et al., 2015).

Another antimalarial 4-aminoquinoline that has been proved against *Leishmania* parasites is amodiaquine. Amodiaquine is a popular antimalarial once used for human treatment, but its use was suppressed by the occurrence of hepatotoxicity due to the metabolization of amodiaquine to form quinone intermediate via hydroxyl oxidation (O’Neill et al., 2003). The amodiaquine has shown an excellent *in vitro* response against intracellular amastigotes of *L. donovani* with a selectivity index (SI) higher than 90 (Guglielmo et al., 2009). Beyond the known antimalarial 4-aminoquinolines (chloroquine, amodiaquine, others), the 4-aminoquinoline scaffold has been widely used as a platform for the construction of new leishmanicidal agents.

We performed a chronological (since 2009) summary of current examples of leishmanicidal 4-aminoquinolines, and from them, a structure–property relationship analysis and the correlation of the biological activity with mitochondrial dysfunction and immunostimulation of host cells were made for those cases where mechanistic data were reported. Furthermore, the specificity of 4-aminoquinolines toward intracellular amastigotes in infected *in vitro* models over promastigote or axenic amastigote *in vitro* models was discussed for most of the cases.

**FIGURE 10:**
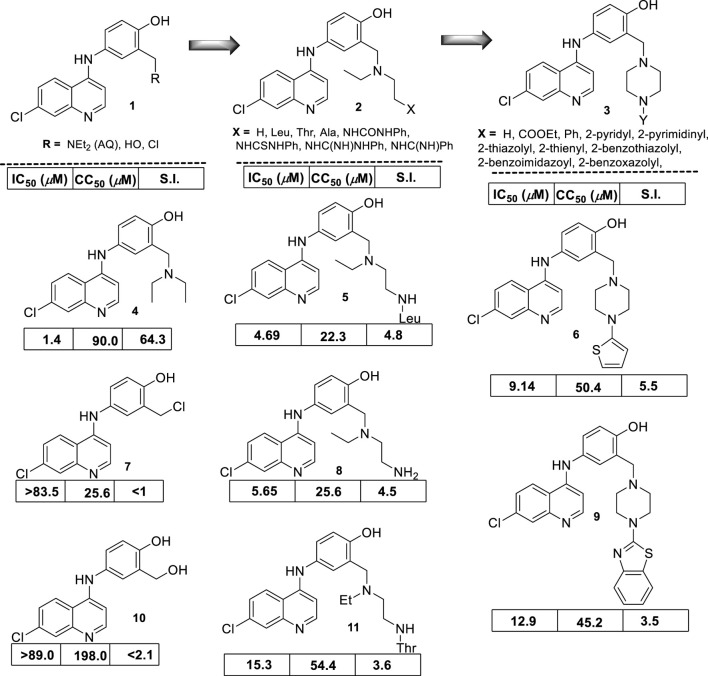
Structure of a series of modified amodiaquine derivatives reported by Bertinaria´s group (Guglielmo et al., 2009). Leishmanicidal response (expressed as IC_50_) against intracellular amastigote *L. donovani* cytotoxicity (expressed as CC_50_) determined on KB cells and selectivity index (SI).

In 2009, S. Gugliemo and co-workers prepared a series of 22 novel analogs of amodiaquine functionalized with an *N*-heteroaryl-piperazine (Guglielmo et al., 2009), a 4-(dialkyl)ethylene-diamine, or an *N*-acyl 4-(dialkyl)ethylene-diamine as the *N*-amine terminal into the 4-aniline moiety (Figure 10), and its leishmanicidal *in vitro* effect against *L. donovani* (responsible of VL) amastigotes was proved. Within the 4-aniline substituted derivatives, the replacement of the terminal *N*-diethylamino in the AQ by hydroxyl-methyl or chloro-methyl moieties resulted in a significant decrease of the leishmanicidal activity for their amodiaquine analogs by more than 80-fold against the intracellular amastigotes of *L. donovani*. This implies that the presence of the basic terminal amine, a pharmacophore, is essential for the design of new amodiaquine leishmanicidal quinolines. Derivatives featuring a 4-(dialkyl)ethylene-diamine, group II, showed an antiamastigote response depending on the type of *N*-substitution at the end of the diamine chain. For example, the incorporation of amine (NH_2_) or leucine amino acid generates the compounds most active in the family (group II), with IC_50_ values of 5.65 µM and 4.69 µM and selectivity indexes of 4.5 and 4.8, respectively. These results show the important role of the terminal amino group, but further improvements are needed to reach the potency found for AQ [selectivity index (SI) of about 90].

The remaining compounds with acyl-amine moieties as terminal substituents showed a similar leishmanicidal activity but higher toxicity, which compromises the selectivity of their analogs. Finally, from group III, the incorporation of heteroaromatic basic moieties was not convenient because their analogs displayed a comparable IC_50_ lower than AQ, but their derivatives were highly toxic, with an SI lower than 3. Only a derivative with a thienyl, a not basic moiety, displayed an acceptable selectivity (SI = 5.5) derived from an IC_50_ of 9.14 µM and a CC_50_ of 50.4 µM, whereas other analogs with a benzothiazolyl moiety showed a selectivity index of 3.5 and an IC_50_ of 12.9 µM and a CC_50_ of 45.2 µM.

From the results, it is clear that the amodiaquine represents a lead platform for the leishmanicidal drug design, and the elongation of the amino chain by the incorporation of an extra group (*e.g*., amino, acyl-amino, or basic heteroaryl) did not improve the leishmanicidal response compared with the amodiaquine standard. No mechanism of action assays were performed.

**FIGURE 11:**
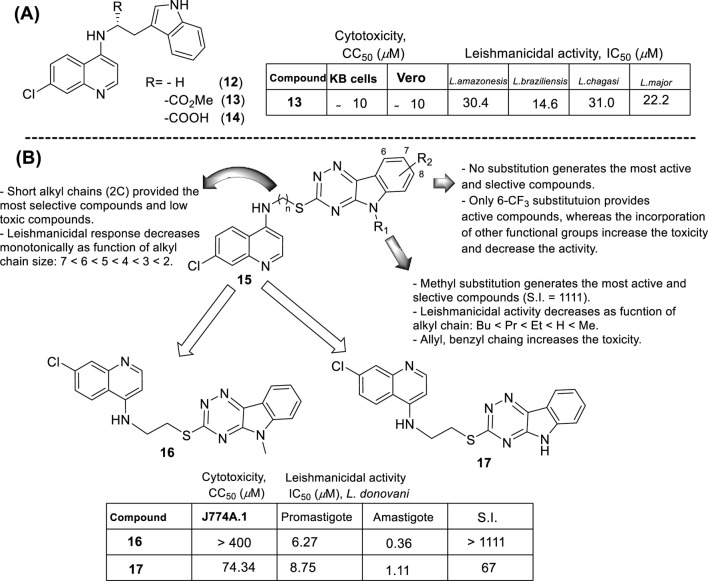
**(A)** Structure of a series of *N*-(2-(indol-3-yl)ethyl)-7-chloroquinolin-4-amines by Coimbra et al. (2010); **(B)** Structure of a series of 7-chloroquinolin-4-amines with a 1,2,4-triazine-benzomidazol fragment reported by P.M.S. Chauhan´s group (Sharma et al., 2014).

In 2010, E.S. Coimbra and co-workers prepared a series of three *N*-(2-(indol-3-yl)ethyl)-7-chloroquinolin-4-amines (compound **11**, **12** and **13**, Figure 11A) (Coimbra et al., 2010), and they were proved against promastigotes of *L. braziliensis, L. amazonensis, L. chagasi*, and *L. major* parasites. In general, these derivatives showed a weak or no leishmanicidal response (IC_50_ > 200 µM) against the different promastigote *Leishmania spp*. In particular, the derivative with the ester moiety as an R substituent (compound **13**) was the most active of the compounds, whereas the compounds with carboxylic moiety (compound **14**) proved to be highly toxic and less selective with an IC_50_ higher than 200 µM and a CC_50_ of approximately 10 µM on Vero and kB cells. These results indicate that a minimal lipophilicity is an essential requirement for the design of a leishmanicidal 7-chloroquinoline featuring a 4-(dialkyl)ethylene-diamine chain. As was described in Section 3, most of the 4-aminoquinolines are usually more active against intracellular amastigotes than promastigotes or axenic amastigotes, which suggests that further experiments of these compounds, in particular, for compound **13**, are required to evaluate the real potential of the hybridized *N*-(2-(indol-3-yl)ethyl)-7-chloroquinolin-4-amines.

In 2013, Chauhan´s group prepared a series of 19 novel quinolines with a triazine indole-thiol alkyl amine at the 4-position (structure **15** in Figure 11B) to be proved against *L. donovani* promastigotes and amastigotes (Sharma et al., 2014). The nature of the hybridization of the triazino indole-thiol-ethylene amino focused on three structural features: (i) the length of the linker (sulfur-alkyl-amino) between the quinoline and the triazino indole; (ii) the *N*-alkyl group incorporated at the indole nitrogen; and (iii) the substitution pattern on the indole’s aromatic ring (C-6 and C-8 position).

From a SAR analysis, the influence of the chain length of the linker, the *N*-indole alkylation, and indole pattern substitution are clear. In general, the short alkyl chain (two carbons, ethylene, compound **16**) provided the most active and selective compounds, finding a monotonic increase of potency and selectivity as a function of the number of carbon atoms as follows: 2C > 3C > 4C > 5C > 6C > 7C > 8C. Interestingly, increasing this carbon chain increases the toxicity of the compounds on J-774A.1 macrophage cells, which compromises the selectivity. Regarding the *N*-alkyl indole substitution, it was found that *N*-methyl substitution provided the most active compound, finding a monotonic increase of leishmanicidal response as a function of *N*-alkyl substitution as follows: *N*-methyl > *N*-ethyl > *N*-propyl > *N*-butyl > *N*-pentyl. *N*-methyl derivative showed an IC_50_ value of 0.36 µM, and the no *N*-substituted hybrid displayed an IC_50_ value of 1.11 µM (compound **17**, Figure 11B), whereas a monotonic increase of IC_50_ was found with an increase of the *N*-alkyl chain length higher than 1 µM. Other nonlinear *N*-alkyl chains, like isopropyl, sec-butyl, allyl, and benzyl, were found to have lower activity levels with IC_50_ values as high as 29.48 µM. Finally, regarding the substitution pattern on the indole’s aromatic ring (C-6 and C-8), except for the introduction of a 6-CF_3_ group (IC_50_ = 6.46 µM), the introduction of any other functional group led to toxic derivatives or derivatives with no leishmanicidal activity (Figure 11B). In summary, Chauhan´s group identified a high selective compound, compound **16**, which presented a high level of activity (IC_50_ = 0.36–7.10 µM) and considerable selectivity indexes (SI from 7 to >1111). Interestingly, the compounds were significantly more selective toward the amastigote form than the promastigote strain.

**FIGURE 12:**
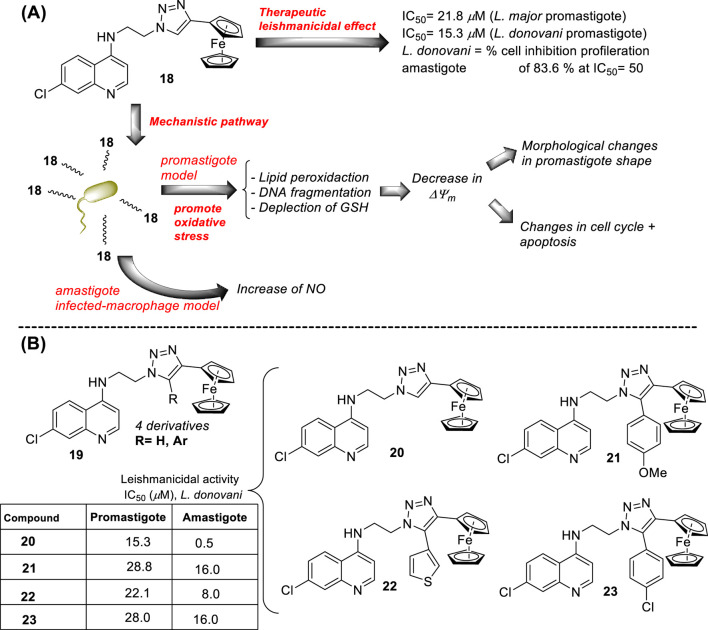
**(A)** Structure of ferrocenylquinoline **18** as a potential antileishmanial agent reported by E.S. Adhikari´s group (Yousuf et al., 2015). **(B)** Structure of ferrocenylquinolines **20–23** as a promising antileishmanial agent reported by Yousuf et al. (2016).

In 2014, S. Adhikari and co-workers prepared a series of 7-chloro-*N*-[2-(1H-5-ferrocenyl-1,2,3-triazol-1-yl)ethyl]quinolin-4-amines (compound **18**, Figure 12A) (Yousuf et al., 2015), and they were proved against *in vitro* models of *L. major* and *L. donovani*. The designed chemical system presents two important structural characteristics: (i) a heteroaryl moiety with basic characteristics at the end of the amino-ethylene chain and (ii) a ferrocenyl moiety that can be interesting due to the role of iron in *Leishmania* recognition. The compound displayed IC_50_ values of 21.86 µM and 15.26 µM against the promastigotes of *L. major* and *L. donovani*, respectively, which was in same range as the leishmanicidal derived from reference miltefosine (IC_50_ = 21 µM) and to other quinoline references such as chloroquine (IC_50_ = 30.00 µM) and ferroquine (IC_50_ = 19.62 µM). The compound exhibited an apparently more significant leishmanicidal response against intracellular amastigotes of *L. donovani* in peritoneal macrophages without cytotoxicity toward murine splenocytes, achieving an inhibition of 83.62% upon treatment of 15 µM (value of IC_50_ determined from the anti-promastigote response against *L. donovani*). The latter revealed that compound **18** was more active against the amastigote form than against promastigotes, which implies that specific targets concerning macrophage-amastigote biological systems could be involved. Further studies showed that compounds promoted changes in the mitochondrial depolarization potential with further biological consequences in promastigote parasites. Scanning electron microscopy (SEM) studies revealed changes in the shape of promastigotes under compound **18** treatment, including loss of flagella and the appearance of porosity in cell membranes with respect to the flagellated and slender non-treated promastigote controls. Flow cytometric analysis showed an increase of the sub-G0/G1 phase from 2.98% (at 6 h) to 9.43% (at 24 h) in comparison with 3.49% in control cells at 24 h. Further assays based externalization of phosphatidylserine residues showed that the percentages of early and late apoptotic cells were increased significantly with respect to untreated cells, finding values of 1.60% and 1.94% (early apoptotic) and of 0.58% and 0.24% (late apoptotic) at 24 h and 48 h, respectively, for untreated promastigotes, and a significant increase under treatment to 12.74% and 23.45% (early apoptotic) and to 18.67% and 24.69% (late apoptotic) at 24 h and 48 h, respectively. Further assays showed that (i) compound **18** promoted a depletion of GSH in a time-dependent manner after 3 h with the highest MFI with respect to the untreated cells; (ii) DNA fragmentation in promastigotes; (iii) increase of ROS level in a time-dependent manner after 3 h of treatment with respect to control culture, (iv) a substantial increase of NO production in comparison with an untreated model of infected macrophages with intracellular amastigotes, and (v) an increase of the levels of lipid peroxides in a time-dependent manner after 1 h and, in particular, after 3 h. With this evidence, the authors proposed that compound **18** acts through an oxidative mechanistic pathway, which is promoted by the presence of the ferrocene moiety in compound **18**. That chemical function induces peroxidation of lipids in the treated promastigotes, which subsequently promotes a decrease in the protein content and changes in the nature of lipidic membranes. This latter induces a loss of mitochondrial membrane potential, leading to apoptosis in *L. donovani* promastigotes. In addition, the release of NO in the infected macrophage model suggests that the compound can also upregulate the innate immune response.

Next, in 2015, following the Adhikari research, Yousuf and co-workers prepared a series of 13 derivatives of ferrocenylquinolines to be proved against *L. donovani* and *L. major* (Yousuf et al., 2016). Four compounds were identified as promising agents against promastigote strains of *L. donovani* AG83 (compounds **19–23**, Figure 12B). Compound **20**, which bears a hydrogen atom as the R substituent (structure **19** in Figure 12B), showed the highest antipromastigote activity, with an IC_50_ value of 15.26 µM, which was more potent than miltefosine (IC_50_ = 21 µM). Compound **20** is the same as compound **18** (Figure 12A), which was studied by Adhikari (Yousuf et al., 2015). Lower antipromastigote responses with IC_50_ values between 22 µM and 28 µM were found for those ferrocenylquinoline derivatives with a heteroaryl/aryl as the R substituent. It revealed that the incorporation of an extra aryl/heteroaryl ring did not favor the leishmanicidal response with respect to the prior compound **18**. Further assays showed that the ferrocenylquinoline **20** was also able to inhibit the growth and proliferation of *L. donovani* LV9 (44.28%) and *L. major* LV39 (52.74%) at 21.8 µM.

Regarding the amastigote stage of *L. donovani* AG83, compound **20** was also able to considerably affect the amastigotes in 50% at 0.5 µM, which supported the specificity of this type of quinolinic compound toward the amastigote form over the promastigote form. Further mechanistic experiments showed, as was described for compound **18**, that this type of compound could arrest cell cycle progression from the increase of sub-G0/G1 cells to 32.88%, 27.31%, and 25.14%, respectively, compared to 2.07% of the control cells after 48 h of treatment. The parasite death was induced by triggering apoptosis. Furthermore, an increase in NO production was recognized from the infected macrophage model under treatment with compound **20**, which opens the door to an immunostimulation role.

**FIGURE 13:**
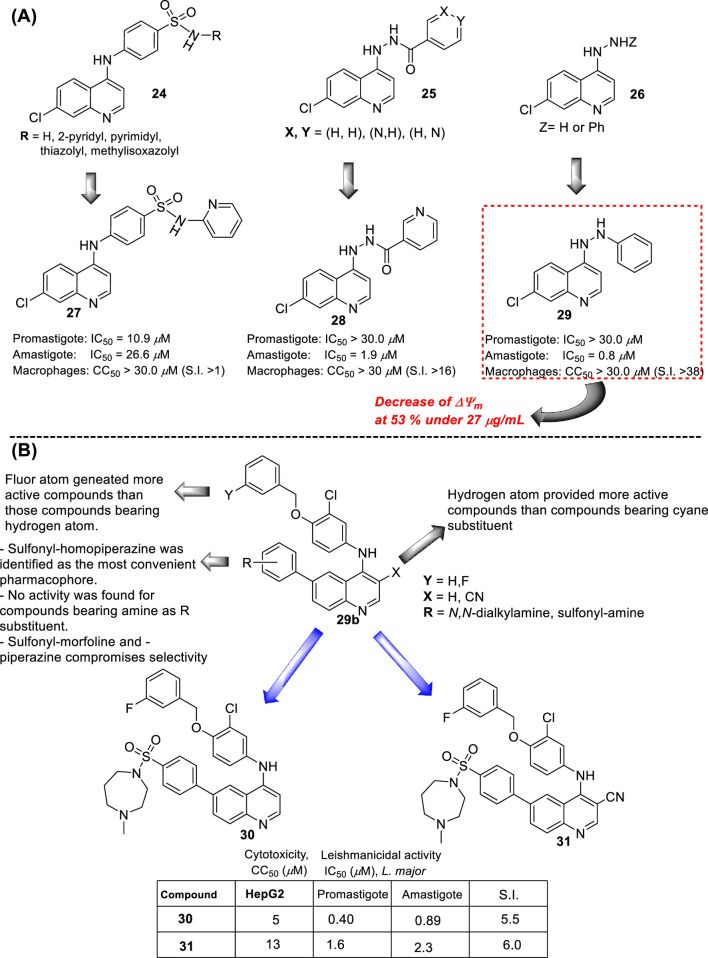
**(A)** Structure of 4-aminoquinolines reported by E.S. Coimbra´s group (Antinarelli et al., 2015). **(B)** Structure of 4-aminoquinolines reported by M. Pollastri´s group (Devine et al., 2015).

In 2015, E.S. Coimbra and co-workers prepared a series of novel ten derivatives of 7-dichloroquinoline functionalized at the 4-position by sulfonamides- (**24**), hydrazide (**25**) or hydrazine (**26**) substitutions (Figure 13A), and the compounds were proved against the amastigote and promastigote forms of *L. amazonensis* (Antinarelli et al., 2015). From sulfonamide derivatives **24**, only those functionalized with pyridine (**27**) displayed good leishmanicidal activity against the promastigote and amastigote forms of *L. amazonensis*, giving IC_50_ values of 10.9 µM and 26.6 µM, respectively. However, its discrete cytotoxicity on macrophages (CC_50_ > 30 µM) compromises its potential by low SI values close to 1.1. Meanwhile, within the hydrazide compounds **25**, only derivative **28** featuring a 3-pyridin heteroaryl exhibited a good antiamastigote response (IC_50_ = 1.9 µM) with a selectivity index of 15.8. Other derivatives consisting of a phenyl or 4-pyridin as the R group in compound **9** generated a discrete anti-amastigote response (IC_50_ between 16.5 µM and 20.7 µM). Finally, quinolin-4-hydrazine and compound **29** exhibited the best antileishmanial activity with IC_50_ of 0.8 µM and 2.4 µM and SI of 37.5 and 12.5, respectively.

A rapid SAR analysis reveals that the incorporation of basic moieties, such as can be seen in structures of groups **24** and **25**, can be an essential pharmacophore for generating active and selective leishmanicidal compounds. Furthermore, the inclusion of a lipophilic moiety into a hydrazine compound (group **26**) could be essential. Interestingly, as seems to be typical in quinoline derivatives, this type of quinoline showed higher activity against the amastigote form than against the promastigote. Mechanistic experiments showed that the compounds can inhibit mitochondrial depolarization potential either in promastigotes or in infected macrophages. For example, a significant reduction in the potential of the mitochondrial membrane in the infected macrophage model was reported with values of 43.6%, 36.7%, and 53.0% under 7.0 μg/mL, 13.0 μg/mL, and 27 μg/mL doses of compound **29**, respectively. The latter was comparable with the depolarization induced by miltefosine (25 μg/mL) of 58%, which is known as a disrupter of membrane potential. The authors found a linear correlation between the anti-amastigote response and the decrease in the *ΔΨ*_*m*_ magnitude.

In the same year, M. Pollastri and co-workers prepared a series of 16 quinolines **29b** with a 4-(benzyloxyl)-aniline and another with a dialkylamine-sulfoxylfenil moieties at the 4- and 6-position (Figure 13B) (Devine et al., 2015). The compounds were evaluated against a diverse type of neglected tropical disease parasite strains, including *T. brucei, T. cruzi, Plasmodium falciparum*, and L*. major*. The quinolinic compounds were divided into two parts: (i) 4- and 6-substituted quinolines and (ii) 4-, 6-substituted 3-cyanequinoline. A consistent SAR analysis was extracted against the *L. major* amastigote parasite. The authors focused their study on the pattern of benzyloxy moiety, quinoline substitution at the 3-position, and the nature of the aryl-substitution at the 6-position. First, the incorporation of benzyloxy-aniline moiety generated the most active compound of the 4-aminoquinolines **29b**. Regarding the benzyloxy moiety, it was found that the fluorine substitution at the 3-position of the benzyloxy ring provides a more active and selective compound than compounds featuring hydrogen atoms. Meanwhile, at the 6-substitution, the sulfonyl-homopiperazine was identified as the most convenient aryl-substitution for the development of the most active and selective compounds. A direct amine (e.g., morpholine) implied a decrease in leishmanicidal activity. Meanwhile, the incorporation of sulfonyl-morpholine and -piperazine aryl-substitution compromises the selectivity in compound **29b**. The inclusion of a cyane moiety at the 3-position of quinoline decreases the activity and selectivity, suggesting that unsubstituted quinolines maintain a significant leishmanicidal response. From the study, two compounds, **30** and **31**, were identified as the most promising compounds, giving IC_50_ values of 0.4 µM and 1.6 µM against promastigotes, respectively, and IC_50_ values of 0.89 µM and 2.3 µM against the amastigote strain, respectively. It implied SI values of 5.5 and 6.0 for compounds **30** and **31**, respectively. No mechanistic exploration was explored for the **29b** type of quinolines.

**FIGURE 14:**
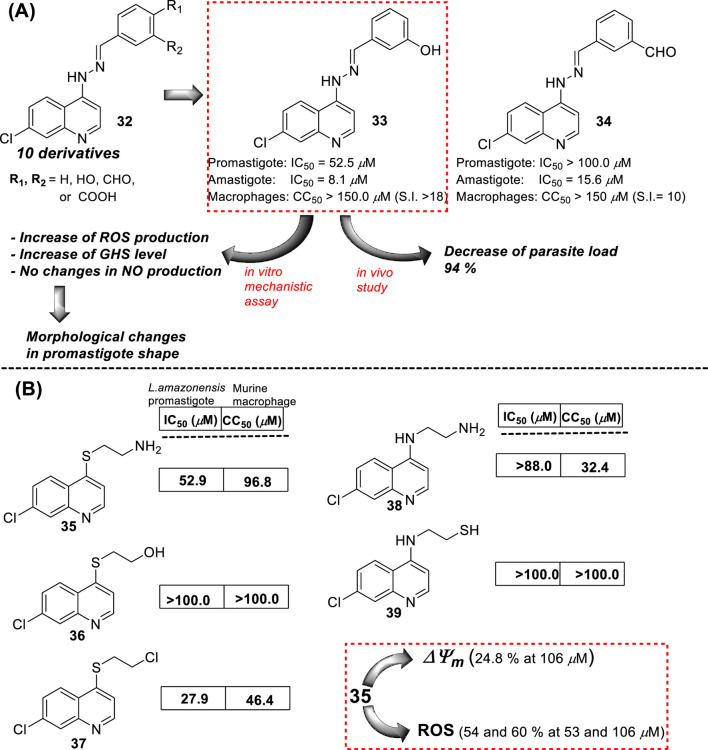
**(A)** Structure of 4-hydrazinylquinoline as an antileishmanial agent reported by E.S. Coimbra´s group (Antinarelli et al., 2016; Antinarelli et al., 2020). **(B)** Structure of 4-amino/sulfur-ethylene-aminequinoline as an antileishmanicidal agent reported by Coimbra et al. (2016).

In 2016, E.S. Coimbra prepared a series of 7-chloroquinolin-4-arylhydrazones **32** and evaluated them against promastigote and amastigote forms of *L. amazonensis* (Antinarelli et al., 2016; Antinarelli et al., 2020) (Figure 14A). Although it is not exactly a 4-aminoquinoline type compound, the existence of the NH-moiety at the 4-position is the key point for our selection. Only two of the ten synthesized compounds displayed an acceptable leishmanicidal response against intracellular amastigotes and low toxicity on murine macrophages (CC_50_ > 150 µM). In particular, compounds **33** and **34** with a 3-hydroxy-phenyl and a 3-formyl moiety, respectively, displayed IC_50_ values of 8.1 µM and 15.6 µM, respectively, which implied selectivity indexes higher than 18 and 10, respectively. Compound **33** was identified as a promising candidate with a better SI (higher than 18) than miltefosine (SI 15.4). Interestingly, those compounds displaying a good antiamastigote activity showed a weak response against promastigotes, which confirmed the high specificity of the quinoline scaffold toward the amastigote stage over the promastigote stage.

Mechanistic studies showed that compounds using compound **33** induced mitochondrial protozoan dysfunction with a dose-dependence, which was in good correlation with a reduction of parasite proliferation in intracellular parasites with intracellular parasite death. Further mechanistic studies reflected that active compound **33** promoted an increase in the ROS levels *in vitro* models of promastigote- and *L. amazonensis*-infected macrophages. In addition, the authors found changes in the level of key factors such as GSH in promastigote models. Through SEM, morphological changes were seen in the parasite shape with loss of flagella compared with flagellated untreated control promastigotes. Further experiments in the amastigote model showed no significant increase in NO production under different concentrations compared with untreated *L. amazonensis*-infected macrophages. This fact suggested that the leishmanicidal activity against the intracellular parasite promoted by compound **33** is possibly more associated with ROS production than immunological activation. *In vivo* studies in the CL model showed that compound **33** led to a significant reduction (48.3%) of cutaneous lesions compared with the control group, finding results comparable to amphotericin reference. Interestingly, the parasitic load was found to be significantly reduced by 93.8% compared to the control group, which was comparable to the parasitic load reduction derived from the AmpB group.

In the same year, E.S. Coimbra presented a series of five 7-chloroquinolines **35–39** featuring an ethylene chain with thiol, amine, or hydroxyl at the beginning or end of the 4-functionalized chain (Coimbra et al., 2016) (Figure 14B), and they were proved against *in vitro* models of *L. amazonensis* (intracellular amastigotes and promastigotes). Only the derivatives **35** and **37**, which bear a 2-aminoethyl-thionyl or a 2-chloroethyl-thionyl chain, respectively, showed a leishmanicidal response with a discrete IC_50_ of 52.9 µM and 27.9 µM against promastigotes of *L. amazonensis*, respectively. Compound **35** exhibited a strong inhibition of the proliferation of *L. amazonensis* amastigotes, giving an IC_50_ value of 0.0911 µM and a selectivity index of 1063. This compound was 139-fold more potent than miltefosine (IC_50_ of 12.7 µM) against amastigotes of the *L. amazonensis* parasite. The high activity against amastigotes and low toxicity on murine macrophages and human erythrocytes were interesting for further assay. Further experiments using compound **35** demonstrated that the compound could promote a decrease in mitochondrial membrane potential and an increase in the ROS level. No substantial NO production in infected macrophages treated with this compound was detected. Compound **35** showed a decrease in the mitochondrial membrane potential (24.8%) only under a high compound concentration (106.0 µM, two times of IC_50_ against promastigotes). Further experiments showed that compound **35** promoted ROS production with percentages of 60.8% and 54.2% under 53.0 µM or 106.0 µM, respectively.

**FIGURE 15:**
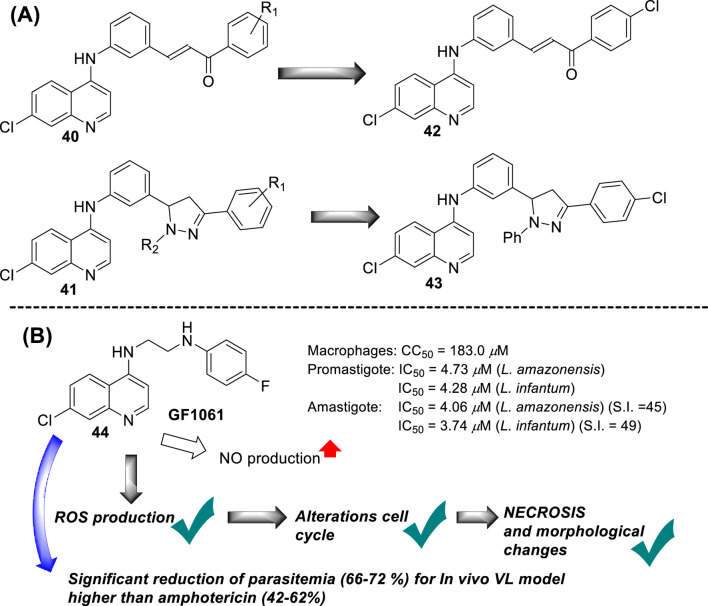
**(A)** Structure of 7-chloroquinolines functionalized at the 4-position by substituted 4,5-dihydro-1H-pyrazole- or chalcone-anilines (Ramírez-Prada et al., 2017). **(B)** Structure of 4-aminoquinoline **44** reported by E.A.F Coelho´s group (Tavares et al., 2019).

In 2017, B. Insuasty and co-workers prepared a series of 37 new derivatives of 7-chloroquinolines functionalized at the 4-position by substituted 4,5-dihydro-1H-pyrazole- or chalcone-anilines (**40** and **41**) (Ramírez-Prada et al., 2017) (Figure 15A), and they were proved against *in vitro* models of *Leishmania panamensis* amastigotes. In general, the quinoline-chalcone derivatives **40** displayed better antileishmanial activity against *Leishmania panamensis* intracellular amastigotes than those quinolines based on 4,5-dihydro-*1H*-pyrazoles **41**. Most of the studied quinoline-chalcones **40** showed good IC_50_ values ranging between 0.79 μg/mL and 2.54 μg/mL. The most active compounds had a 4-chloro, 4-bromo, or 4-methyl substitution in the aryl moiety. However, they were highly cytotoxic with low CC_50_ values of approximately 0.7–2.0 μg/mL on promonocytic human cell U-937 cells. Meanwhile, among the quinoline-based 4,5-dihydro-*1H*-pyrazoles **41**, although their derivatives displayed lower leishmanicidal response than quinoline-chalcones, their derivatives displayed acceptable SI values of about 17.1, 14.9, 16.6, and 72.2 for some derivatives including (R_2_ = Ph, R_1_ = 4Cl), (R_2_ = Ph, R_1_ = 3,4,5-(OMe)_3_), (R_2_ = 3,4-DiClPh, R_1_ = 4Cl), and (R_2_ = 3,4-DiClPh, R_1_ = 4Br) as consequence of their low cytotoxicity. Then, the leishmanicidal activity of the quinoline-based 4,5-dihydro-*1H*-pyrazoles was dependent on the nature of the R_1_ and R_2_. The R_1_-substitution was essential to generate active compounds, where the phenyl moiety provided more active compounds than those derivatives with formyl or acetyl moieties, which implies the importance of the incorporation of a highly lipophilic moiety like phenyl. Meanwhile, the R_2_ substitution allowed modulating the level of biological activity, finding the most active and selective response for the R_1_-substitution of 4-bromo-, 4-chloro-, or, eventually, 3,4,5-trimethoxy-. Importantly, none of the compounds presented an IC_50_ lower than amphotericin B (0.05 μg/mL). From both chemical platforms **40** and **41**, compounds **42** and **43** were identified as the most active and selective of the compounds, respectively, although they were sufficiently active for further assays. No mechanistic assays were reported by the authors.

In 2018, E.A.F Coelho’s group prepared compound GF1061 (compound **44**) from the coupling between the *N*-(2-bromoethyl)-7-chloroquinolin-4-amine with an excess of 4-fluoroaniline in the presence of potassium carbonate in DMF under reflux. The compound was proved against *in vitro* models of *L. amazonensis* and *L. infantum*. GF1061 showed a good leishmanicidal response with EC_50_ values of 4.73 μM and 4.28 μM against promastigotes of *L. amazonensis* and *L. infantum* parasites, respectively (Tavares et al., 2019) (Figure 15B). Against axenic amastigotes, GF1061 displayed EC_50_ values of 4.06 μM and 3.74 μM against *L. amazonensis* and *L. infantum*, respectively. AmpB presented IC_50_ values of 0.13 μM and 0.09 μM against *L. amazonensis* and *L. infantum* promastigotes, respectively, and of 0.39 μM and 0.30 μM against their amastigote strains, respectively. Despite the higher biological response of amphotericin to compound **44**, the quinoline compound displayed a lower toxicity (CC_50_ of 183.0 μg/mL) than amphotericin (57.64 μg/mL), which implied SI values of 45.0 and 48.9 against the amastigotes, respectively, which was superior to SI values of 2.2 and 2.7 found for amphotericin. Regarding the study against intracellular amastigotes, a significant reduction in infection was found for infected macrophages treated with GF1061, finding % infection lower than 25% for a GF1061 concentration of approximately 2.5 μM. That higher leishmanicidal response against intracellular than axenic forms of the parasite confirms the high preference of the 4-aminoquinoline scaffold toward clinically relevant amastigotes in infected models. Further assay showed that GF1061 promoted a significant reduction in the mitochondrial membrane potential (*Δψ*_*m*_) in the *in vitro* promastigote model of *L. infantum*, giving values of 34.3% and 34.5% upon compound concentrations of 2.98 μg/mL and 5.96 μg/mL, respectively. Further assay showed that compound **44** induced an increase in the levels of intracellular ROS in promastigote parasites upon the action of the compound at 6 h or 24 h. Analysis of parasite membrane integrity showed that the treatment with GF1061 induced significant morphological alterations in *Leishmania* promastigotes. Further evaluations showed that alterations in the parasite cell cycle were found in the G0/G1 phase for the treated parasites, which suggests the ability of the compound to promote DNA fragmentation. The cell integrity alterations were interpreted from the significant reductions in the G1 and G2 phases in treated parasites.

Further assays based on phosphatidylserine exposure in the cell surface showed the absence of an apoptotic process in the GF1061-treated parasites with strong evidence of death via necrosis. No substantial NO production in infected macrophages treated with this compound was detected. The leishmanicidal response of compound **44** could be associated with the depolarization of mitochondrial potential, which promoted ROS production that subsequently induced alteration in the cell cycle by death via necrosis with morphological consequences. Finally, compound **44** showed a significant *in vivo* efficacy for a VL model, finding an appreciable reduction in parasitemia in the infected tissue, liver, spleen, and dLN, giving a percentage of parasitemia reduction of 66%, 69%, 71%, and 72%, respectively, which was comparable with results found under the amphotericin drug of 62%, 44%, 38%, and 48%, respectively. Compound **44** emerged as a potential candidate as a leishmanicidal agent with an appreciable effect of depolarization on mitochondrial potential as the main mechanism of action.

**FIGURE 16:**
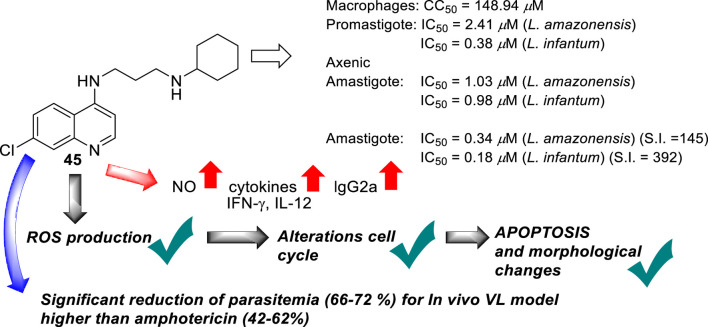
Structure of 4-ethylenediamine quinoline as a potential antileishmanicidal agent reported by E.S. Coimbra´s group (Sousa et al., 2019).

In 2019, Coelho’s group prepared a new type of 4-aminoquinoline called AM1009, compound **45**. That compound was synthesized through a direct reaction between the *N*-(3-bromopropyl)-7-chloroquinolin-4-amine and an excess of cyclohexanamine in the presence of potassium carbonate under reflux (Sousa et al., 2019) (Figure 16). AM1009 was evaluated *in vitro* against *L. amazonensis* and *L. infantum* promastigotes and their axenic amastigotes. AM1009 showed a good antileishmanial response with EC_50_ values of 2.41 μM and 0.38 μM against the promastigotes of *L. amazonensis* and *L. infantum*, respectively. Against axenic amastigotes, AM1009 displayed EC_50_ values of 1.03 μM and 0.98 μM against *L. amazonensis* and *L. infantum*, respectively. AM 1009 showed lower leishmanicidal activity than amphotericin drug (0.13 μM and 0.09 μM against *L. amazonensis* and *L. infantum* promastigotes, respectively, and of 0.34 μM and 0.18 μM, respectively), but interestingly, AM1009 was less toxic on murine macrophages than amphotericin (CC_50_ value of 148.94 μM vs. 0.85 μM), which also implies better SI values of 144.6 and 392.0 against axenic amastigotes of *L. amazonensis* and *L. infantum*, respectively. Amphotericin displayed SI values of 2.5 and 4.7. Importantly, AM1009 showed lower toxicity against red blood cells with an RBC_50_ value of 1739.69 μM. Against the infected model of macrophages, AM1009 displayed a reduction of infection higher than 50% for concentrations of 0.18 and 0.34 μM against models of *L. infantum* and *L. amazonensis*, respectively, which are in same range to IC50 values found for the amphotericin drug against these infected models. Mechanistic assays revealed that AM1009 induced a significant decrease of mitochondrial membrane potential at 2 μM (24.6%) against promastigotes of *L. amazonensis*, which was comparable to that found for the FCCF reference (35.9%). These results were in good concordance with a higher production of ROS and significant alterations in their cell cycle by the occurrence of cells in the sub-G0/G1 phase and the formation of autophagic vacuoles promoted on *L. amazonensis* promastigote parasites treated with AM1009. All these results indicate cell apoptosis promoted by AM1009. A further assay based on immunological studies showed high levels of the cytokines IFN-γ, IL-12, and GM-CSF, a significant increase of nitrite ions, and an increase in levels of the parasite-specific IgG2a isotype antibody. These results suggested that AM1009 could be acting as an immunostimulating agent, favoring the development of polarized Th1 response, which increases the levels of essential citoquines and NO. In addition, in the parasite, the compound promoted depolarization of mitochondrial potential, which induces the increase of ROS, and, subsequently, changes in the cell cycle. Therefore, AM1009 could have a double action, an immunostimulant activity and a leishmanicidal action derived from alteration in mitochondria functions. Finally, for a murine model of CL infected with *L. amazonensis*, AM1009 achieved a significant decrease in the size of the lesion and parasite load in infected mice, with a better efficacy than amphotericin.

**FIGURE 17:**
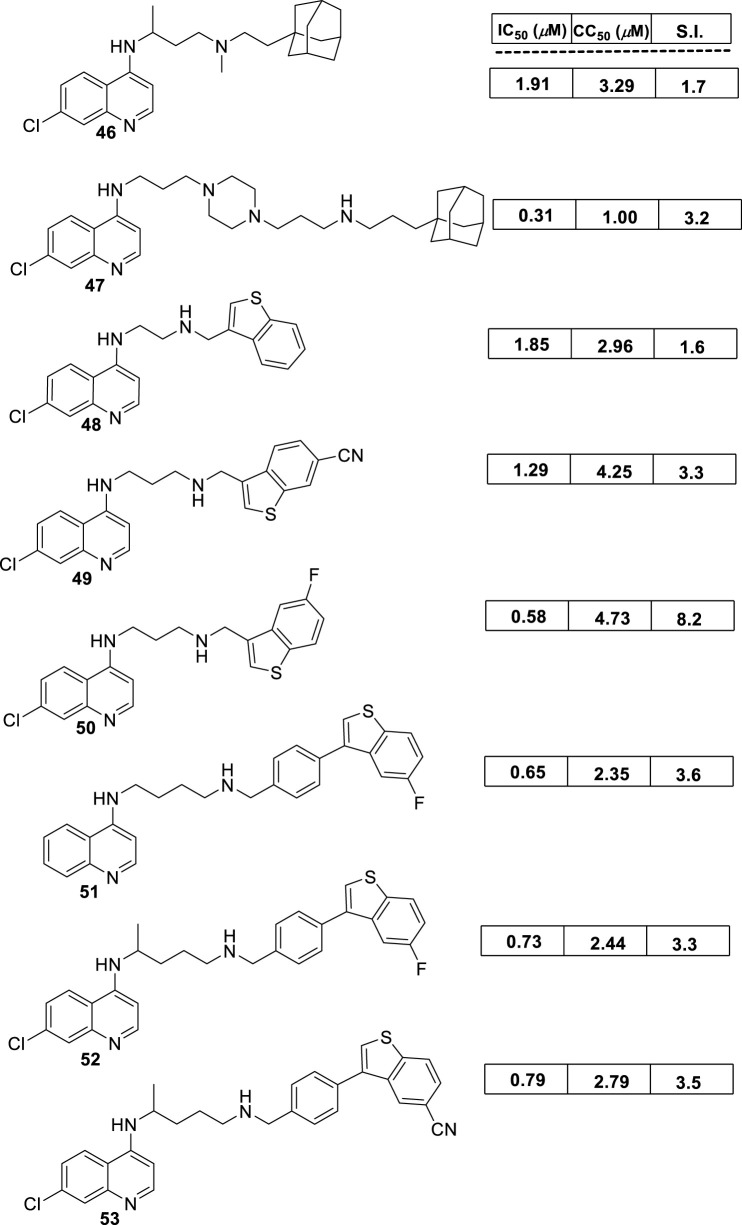
Structure of complex 4-aminoquinolines functionalized through diamine-alkyl chain reported by B.A. Solaja´s group (Konstantinović et al., 2018).

In 2018, Bogdan A. Solaja’s group designed and synthesized a series of 30 7-chloroquinolines featuring alkyl-diamino chains at the 4-position with different structural characteristics involving the incorporation of aryl- or rigid alkyl groups at the end of the alkyl-diamino chain (Konstantinović et al., 2018) (Figure 17). Furthermore, other chemical functions at the 3- and 7-positions were incorporated into synthesized compounds. The compounds were divided into seven groups: (i) group I consisting of chloroquine analogs functionalized at 3-position by nitro and amino moieties, (ii) group II consisting of chloroquine analogs with an adamantino moiety at the end of the alkyl-diamine chain, (iii) group III consisting of chloroquine analogs with a benzothiazole moiety at the end of the alkyl-diamine chain, (iv) group IV consisting of chloroquine analogs with a phenyl-3-benzothiazole moiety at the end of the alkyl-diamine chain, (v) group V consisting of tetrahydroquinoline with a diethylamino moiety at the end of the alkyl-diamine chain like chloroquine, (vi) group VI consisting of tetrahydroquinoline with a (4-cyanephenyl)-2-thiophenyl-4-fenil moiety at the end of the alkyl-diamine chain, and (vii) group VII consisting of chloroquine with a (4-cyanophenyl)-2-thiophenyl-4-fenil moiety at the end of the alkyl-diamine chain. A structure–property relationship analysis showed compounds from group II to group VII showed a sub-micromolar response against *L. infantum* and *Leishmania tropica* promastigotes (0.3–4.0 µM), whereas the compounds of group I showed a weaker response (4.0–10.0 µM). From the antipromastigote response, no significant differences in biological activity were found between compounds featuring an ethylene-diamine chain and compounds with a propylamine chain. No significant changes in antipromastigote response were found by the suppression of the 7-chloro substitution by hydrogen. Meanwhile, the functionalization at the end of the alkyl-diamine chain leads to appreciable changes in the leishmanicidal response. From this QSAR relationship, eight compounds, **46–53**, from group II through group VII were chosen as the most promising compounds for an *in vitro* evaluation against *L. infantum* and *L. tropica* promastigotes (Figure 17), and further *in vitro* evaluations against intracellular amastigotes of *L. infantum* parasite were performed. A comparison between selected candidates revealed that the compound with the (4-cyanophenyl)-2-thiophenyl-4-fenil **53** moiety (0.6–0.8 µM) was the most active, followed by those with a benzothiazole (1.0–1.4 µM), whereas a derivative of adamatino showed a significant IC_50_ of 0.31 µM. Results showed that lipophilicity must be considered, but it seems important that elongated alkyl-diamine chains are not convenient because although active compounds are generated, they promote more side effects on the host cell.

Solaja selected compounds **47** and **50** with selectivity indexes of 3.2 and 8.2, respectively, for further *in vivo* evaluation for a model of VL. Interestingly, the authors found a significant *in vivo* response, finding a significant reduction of liver parasitemia by about 99%, using a low compound dose of 5 mg/kg. Despite the results, further toxicity assays are needed to confirm the potential of the compounds as lead candidates. No mechanistic experiments were reported. This report put in evidence the structural relevance of the incorporation of a weak basic moiety like an alkyl-diamine chain with a tertiary amine as well as the incorporation of a lipophilic moiety composed by an aryl-thiophenyl or an adamant moiety.

**FIGURE 18:**
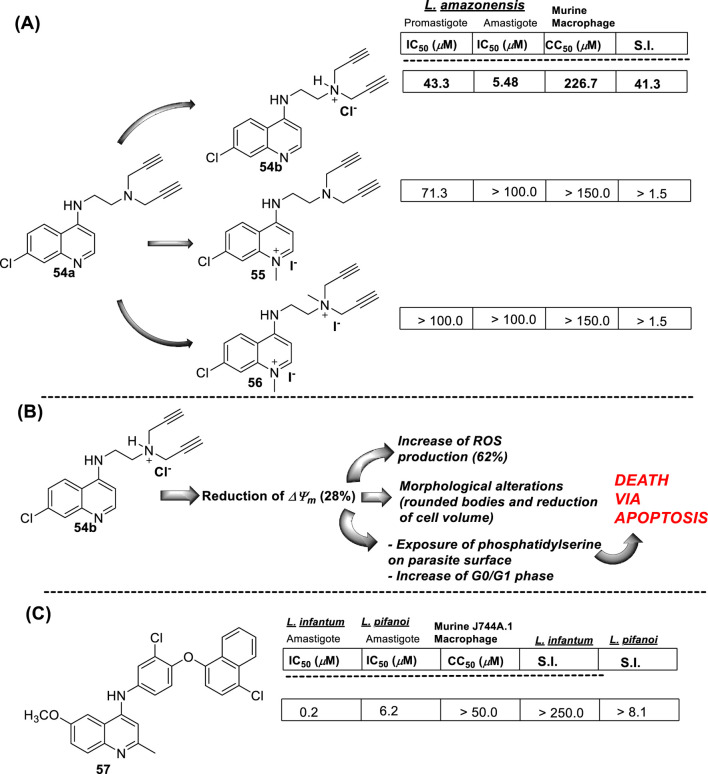
**(A)** Leishmanicidal *in vitro* activity of 4-aminoquinolinic salts with terminal 3-propinyl reported by Calixto et al. (2018). **(B)** Mechanistic data derived from compound **54b**. **(C)** Structure of 6-methoxy-quinalidine **57** reported by Stevanovic et al. (2018).

In 2018, Calixto and co-workers synthesized a series of organic salts from an *N* ^*1*^-(7-chloroquinolin-4-yl)-*N* ^*2*^ *,N* ^*2*^-di(prop-2-yn-1-yl)ethane-1,2-diamine **54a-b, 55**, and **56** (Calixto et al., 2018) (Figure 18A), and they were evaluated against *in vitro* models of *L. amazonensis*, and *L. braziliensis*. The 4-aminoquinoline **54a** previously showed a discrete activity against different *Leishmania* parasites for *in vitro* models (Carmo et al., 2011). The new organic salts were based on the *N*-alkyl protonation of 4-aminoquinoline **54a**, the *N* ^*1*^-methylation, and the double action, *N* **-**alkyl protonation, and *N* ^*1*^-methylation, to give the corresponding water-soluble compounds **54b**, **55**, and **56**, respectively. This cationization seeks to improve the leishmanicidal response through an improvement of the physical-chemical properties. The three cationic compounds were evaluated for their leishmanicidal response against *L. amazonensis* and *L. braziliensis* promastigotes and amastigotes. The results of the *in vitro* evaluation against promastigotes demonstrated that only the derivative **54b** was effective against both species of *Leishmania* (IC_50_ [*L. amazonensis*] = 43.25 µM and IC_50_ [*L. braziliensis*] = 39.19 µM). Furthermore, only compound **54b** was active against *L. amazonensis*-GFP intracellular amastigotes (IC_50_ = 5.48 µM), and that antiamastigote activity was very similar to that observed against *L. amazonensis*-Wild type amastigotes (IC_50_ = 5.62 µM). Importantly, compound **54b** presented a low level of toxicity against murine macrophages (IC_50_ = 226.70 µM), making compound **54b** a promising candidate by its higher SI (by about 40 units) against *L. amazonensis* amastigote strains. Further experiments confirmed the ability of compound **54b** to induce oxidative stress via depolarization of mitochondrial membrane potential. Compound **54b** induced a reduction of *∆Ψ*_*m*_ of 28% at a compound concentration of 86 µM. Interestingly, the compound displayed a reduction of mitochondrial potential comparable with that displayed under miltefosine, and no effect on the mitochondrial potential was observed for uninfected macrophages under treatment with compound **54b**. On the other hand, compound **54b** at 86 µM in culture promoted an increase of ROS production by about 62%, which was similar to miltefosine (68%) (Figure 18B). In addition, TEM revealed that compound **54b** promoted some morphological modifications such as rounded bodies and reduction of cell volume. To analyze the reason for death, TEM showed that promastigotes, upon treatment with compound **54b**, induced exposure of phosphatidylserine on the parasite surface, reflecting an increase of the cell population in the sub-G0/G1 phase with condensed and marginalized chromatin, suggesting death via apoptosis.

In addition, in 2018, S. Stevanovic’s group identified a substituted 6-methoxy-quinalidine **57** (Figure 18C) from a virtual screening of the *L. infantum* type 2 NADH dehydrogenase (NDH2) for a group of ubiquinone analogs including a 4-aminoquinoline, phthalazine, quinolone, and other *N*-heteroarenes (Stevanovic et al., 2018). The quinolinic compound **57,** as well as other *N*-heteroarenes ubiquinone analogs, were evaluated *in vitro* against *L. infantum* axenic amastigotes and promastigotes. Compound **57** exhibited an excellent leishmanicidal response against the wild type of *L. infantum*, giving IC_50_ values of 0.03 and 0.2 µM against promastigotes and amastigotes, respectively. Later, Rivas’s group reported a good response against *L. donovani* promastigotes and *Leishmania pifanoi* amastigotes, giving IC_50_ values of 4.4 µM and 6.2 µM, respectively. Rivas´s group also reported a moderate cytotoxicity with a CC_50_ value higher than 50 µM using J774A.1 macrophages, which implies SI indexes higher than 250 and 8.1 for *in vitro* amastigote models of *L. infantum* and *L. pifanoi*, respectively. This report is an interesting finding because it showed that the incorporation of an extra basic alkyl chain is needed to generate a potent and selective compound, although it cannot be discarded if the *N*-alkyl chain could increase the leishmanicidal effect even more.

**FIGURE 19:**
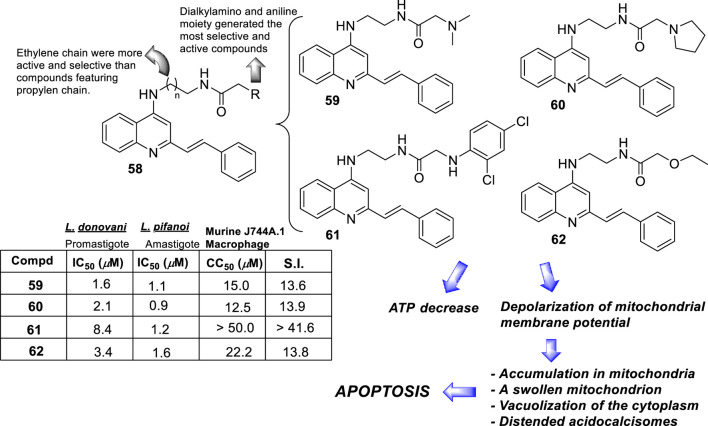
Structure of 4-amino-2-styrylquinolines **58** and their most active derivatives **59–62** reported by Rivas´s group (Staderini et al., 2019).

Based on the widely known potential of quinoline derivatives as antileishmanial agents, another research group designed a new class of 4-amino-2-styryl quinolines **58** and evaluated them against *L. donovani* promastigotes and *L. pifanoi* amastigotes (Staderini et al., 2019) (Figure 19). Furthermore, some other derivatives were also synthesized and evaluated to serve as a control group, allowing a deeper understanding of the structural influences of the 4-amino-2-styryl quinolines. Considering their effects against *L. donovani* promastigotes, the results demonstrated that, except for 2-styrylquinoline (not bear 4-substitution), all the evaluated molecules with a 4-*N*-alkyl chain present considerable leishmanicidal properties, giving IC_50_ values ranging from 0.2 µM to 35.1 µM. Interestingly, most of the evaluated compounds were more potent, making it possible to verify the importance of a 2-styryl group, whose remotion led to a considerable loss of leishmanicidal activity. Four compounds **59–62** were chosen as promising candidates for their good leishmanicidal profile (Figure 19). From the leishmanicidal response, compounds displayed a higher activity against the amastigote strain than against the promastigote strain. Against *Leishmania* amastigotes, compounds presented low IC_50_ values from 0.9 µM to 1.6 µM for compounds **59–62**. In particular, compound **61** exhibited the highest SI value, giving a magnitude higher than 42. The compound is characterized by an aniline as terminal moiety on the *N,N*-dialkyldiamine chain, which seems to be more convenient to increase the selectivity than dialkyl amine or methoxy as terminal moieties on the *N,N*-dialkyldiamine chain.

Beyond the leishmanicidal effect, the authors explored the mechanism of action based on mitochondrial dysfunction. First, the author studied the levels of intracellular ATP upon compound treatment using *L. donovani* promastigotes from the 3-Luc strain. This parasite expresses a cytoplasmic form of firefly luciferase that is episomally encoded. At 50 mM compound concentrations, most of the studied compounds **58** induced a decrease in luminescence, which supports the decrease in ATP synthesis. To demonstrate that the drop in ATP production is associated with permeabilization, the authors explored the permeabilization by the addition of triton X-100, finding no alteration of the plasma membrane permeability. Alternatively, the authors studied the effect of these compounds on membrane mitochondrial potential, finding a decay of *ΔΨ*_*m*_ compared to negative controls. Accumulation into mitochondria using mitotracker supports the role of the parasite mitochondria as a target for compounds **58**, which was demonstrated through confocal microscopy. A TEM study on *L. donovani* promastigotes showed three key morphological characteristics: (i) a swollen mitochondria, (ii) strong vacuolization of the cytoplasm, and (iii) the appearance of distended acidocalcisomes. This evidence reflects the role of the mitochondria as key targets for promoting apoptosis and also provides evidence for the role of other organelles like acidocalcisome as targets to store divalent Ca^2+^ and heavy cations, Fe^2+^ and Zn^2+^, which could be able to capture quinoline compounds via coordination binding.

**FIGURE 20:**
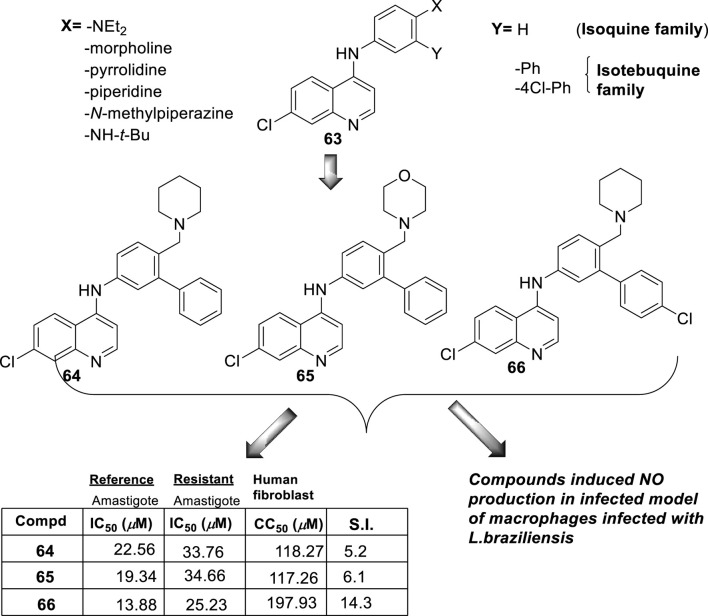
Structure of isoquine and isotebuquine derivatives **63–66** reported by Romero et al. (2019).

In 2019, we reported the activity of a series of dehydroxylated derivatives of isoquine and isotebuquine **63** against *in vitro* models of *L. braziliensis and L. Mexicana* (Figure 20) (Romero et al., 2019). These derivatives previously displayed a good antimalarial response with curative properties for an *in vivo* model of *Plasmodium berghei* (Romero et al., 2015; Valverde et al., 2017). Regarding *Leishmania* parasites, interestingly, these derivatives, dehydroxilated from isotebuquine, showed a higher leishmanicidal response than their isoquine analogs either against *L. braziliensis* promastigotes and *L. mexicana* parasites (Romero et al., 2019). The latter revealed that the incorporation of the extra phenyl ring on the aniline moiety was essential for promoting a significant leishmanicidal response. Three of the 12 evaluated compounds (**64**, **65**, and **66**) showed a promising response for further studies. From cytotoxic assay, derivatives **64**, **65**, and **66** showed good SI values against *L. braziliensis* of 11.4, 14.1, and 52.1 and of 17.9, 18.3, and 24.1 against *L. mexicana*, respectively. Against amastigote strains, compound **64** showed better antiamastigote response (IC_50_ = 13.88 µM) than compound **65** (IC_50_ = 22.56 µM) and compound **66** (IC_50_ = 19.34 µM), which implied SI values of 14.3, 6.1, and 5.2, respectively. The antiamastigote response of compound **65** was barely higher than glucantime (IC_50_ = 15.12 µM). Interestingly, compound **66** showed the best antiamastigote response against a resistant strain of *L. braziliensis,* giving an IC_50_ value of 25.23 µM (SI 7.8), higher than that found by using glucantime (IC_50_ > 50 µM). From a mechanistic point of view, a discrete increase of NO was found from an intracellular amastigote model with a concentration dependence, which suggested a specific effect against infected macrophages that could be associated with an immunostimulation response. Currently, we are studying the potential of these derivatives against *in vitro* models of *L. infantum*, finding excellent responses against infected models in sub-micromolar ranges.

**FIGURE 21:**
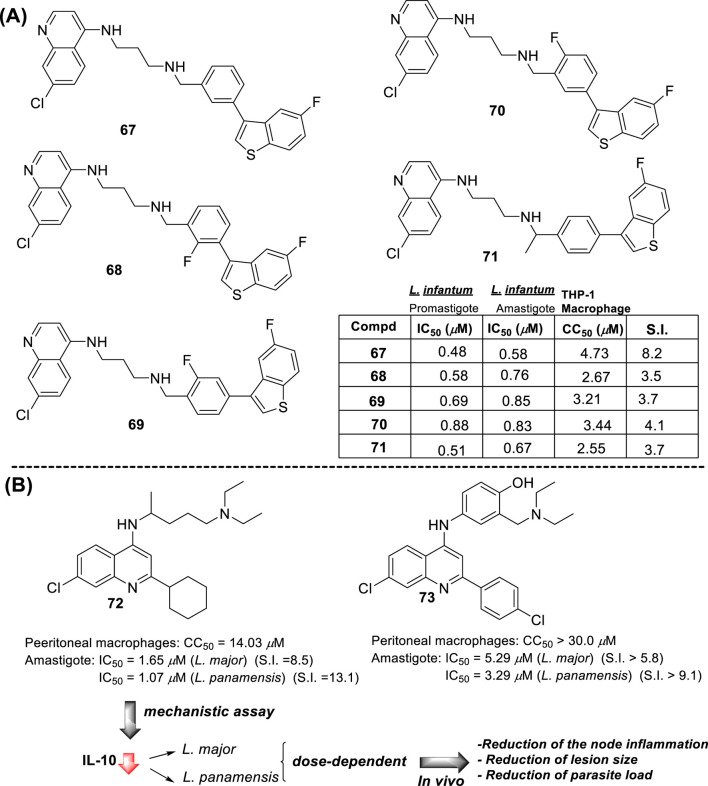
**(A)** Structure of complete 4-aminoquinoline hybrid functionalized through diamine-alkyl with 2-benzothiazol-benzyl-amine moiety reported by Manzano et al. (2019). **(B)** Structure of a chloroquine **72** and an amodiaquine **73** analog with a lipophilic moiety at the 2-position of quinoline core, reported by *P. Fernandez* (Herrera et al., 2016; Herrera et al., 2020).

In 2019, N. Basilico, F. Gamarro, and co-workers development a series of five quinoline hybrids **67–71** as depicted in Figure 21A (Manzano et al., 2019). This research is a continuation of a previous report (Konstantinović et al., 2018). All compounds exhibited significant antipromastigote and antiamastigote responses, giving IC_50_ values lower than 1 µM (Figure 21A), finding similar leishmanicidal responses between promastigote and amastigote strains. In particular, among the five compounds, compound **67** showed the best SI profile with an SI value of 8.2, whereas the remaining compounds displayed SI values between 2 and 4. A small superficial structure–activity analysis reflects that the location of the 2-benzothiazol at the 3-position of the benzylic-amine is preferred over the location at the 4-position in order to decrease the cytotoxic effect and achieve a higher SI. It is important to mention that the leishmanicidal effect is similar among all studied compounds. On the other hand, mechanistic experiments demonstrated that compound **67** promoted (i) an alteration in the energetic metabolism with a significant loss of ATP that is associated with the depolarization of mitochondrial potential (*ΔΨ*_*m*_), (ii) a decrease of intracellular ATP levels with an alteration of plasma membrane permeability, and (iii) a significant ROS production, which is the typical mode of action found for 4-aminoquinolines. This type of mechanism initiates a depolarization of mitochondrial membrane potential, which subsequently promotes ROS production that promotes alterations in parasite morphology with apoptotic consequences as the mode of cell death.

In 2020, *P. Fernandez* synthesized and evaluated the leishmanicidal activity of two 7-chloroquinoline derivatives **72** and **73**. Compound **72** displayed IC_50_ values of 1.65 and 1.07 against intracellular amastigotes of *L. major* and *L. panamensis*, respectively, whereas compound **73** displayed IC_50_ values of 5.29 and 3.29 µM against *L. major* and *L. panamensis*, respectively (Herrera et al., 2016; Herrera et al., 2020) (Figure 21B). Compounds showed CC_50_ values of 14.03 μM and higher than 30 μM, respectively, which implied SI values of 8.5 and 13.1 for compound **72** and 5.8 and 9.1 for compound **73**, against *L. major* and *L. panamensis*, respectively. Interestingly, neither compound showed a response against promastigotes at 50 µM (IC_50_ > 50 µM), which supports the high specificity of this type of quinolines toward the amastigote form over the promastigote form. Further assays showed the tentative immunostimulating response of these compounds. In particular, compound **72** inhibited the production of the pro-inflammatory cytokine of IL-10 by macrophages infected with *L. panamensis* and *L. major* in a dose-dependent manner. These results suggest that the compound induced a parasite-killing mechanism through regulation of the macrophage activation, as was explained in Section 2.3.

Further studies demonstrated the efficacy of compound **72** on a murine model of *L. panamensis* (Herrera et al., 2020). The compound promoted an appreciable reduction of the node inflammation, lesion size, and parasitemia index at the lesion region in a dose-dependent manner. In addition, the authors found a significant reduction in the IL-10 production from the lymph node cells of infected mice. Then, it seems that the incorporation of either a basic moiety or a lipophilic moiety is essential for the development of selective and potent leishmanicidal agents based on 4-aminoquinoline.

**FIGURE 22:**
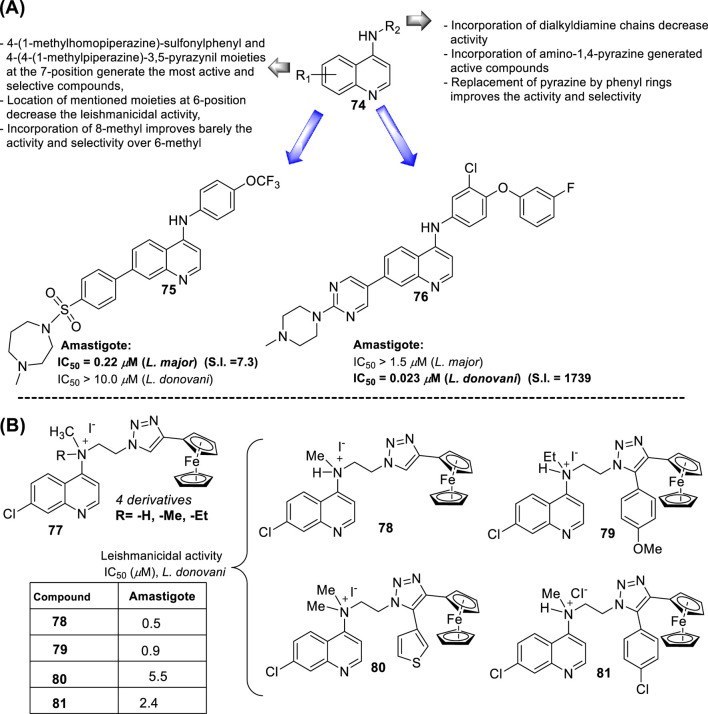
**(A)** Structure of 4-aminoquinolines **74–76** reported by L. Ferrins-M. Pollastri (Singh et al., 2020). **(B)** Structure of flexible and water-soluble ferrocenylquinoline *N*-quaternized compounds **78–81** (Mukherjee et al., 2020).

At the beginning of 2020, L. Ferrins and M. Pollastri followed a target repurposing and parasite-hopping approach and developed a series of quinoline derivatives originating from the reoptimization of lapatinib to NEU-1953 and further optimizations of this (Ferrins et al., 2018; Mehta et al., 2018; Bachovchin et al., 2019). This entire series of quinoline derivatives was then evaluated for its leishmanicidal potential against *L. major* and *L. donovani* intracellular amastigotes (Singh et al., 2020). Focusing on the *in vitro* leishmanicidal effect against intracellular amastigotes of *L. major* parasite, many of the designed quinoline hybrid **74** compounds displayed a remarkable leishmanicidal activity (Figure 22A). A SAR analysis showed that the nature of the R_1_ and R_2_ substituents is pivotal for leading an active and selective compound. Talking about the R_2_ substitution, the incorporation of dialkyl amine chains decreased the leishmanicidal activity, whereas the inclusion of amino-1,4-pyrazine generated active compounds. More improvement was found when the pyrazine moiety at the 4-position was replaced by composed phenyl rings. In particular, the substituted anilines such as 3′-chloro-4′-methoxyaniline **75** and 4-(trifluoromethoxy)aniline **76** generated the most active compounds (Figure 22A).

Meanwhile, regarding the R_1_ substitution, the 4-(1-methyl homopiperazine)-sulfamoylphenyl and 4-(4-(1-methylpiperazine)-3,5-pyrazinyl moieties at the 7-position generate the most active and selective compounds, whereas its location at the 6-position decrease the leishmanicidal activity. Interestingly, the introduction of methyl-groups at different positions of the quinoline core provides different effects. 6-Methylation improves the leishmanicidal effect, whereas an 8-methylation promotes a loss of activity. From the proved compounds, compounds **75** and **76** were chosen as promising candidates for their high antiamastigote response against infected models of *L. major* and *L. donovani* macrophages. Compound **75** was more active against *L. major* (IC_50_ = 0.22 µM, SI = 7.3) than against *L. donovani* (IC_50_ > 10.0 µM). In contrast, compound **76** was more active agains*t L. donovani* (IC_50_ = 0.023 µM, S.I = 1739 using HepG2 cell as reference) than against *L. major* (IC_50_ > 1.5 µM). Compound **76,** with an SI of 1739, represents a potential candidate for further evaluation of either mechanistic or *in vivo* efficacy.

In 2020, S. Adhikari-C.Pal and co-workers prepared a series of novel flexible and water-soluble ferrocenyl quinoline *N*-quaternized compounds **77** (compounds **78–81**, Figure 22B) (Mukherjee et al., 2020). From an *in vitro* evaluation against intracellular amastigotes of *L. donovani*, compound **78** displayed the best leishmanicidal response, giving an IC_50_ value of 0.50 μM, whereas compounds **79**, **80**, and **81** displayed IC_50_ values of 0.99 µM, 5.05 µM, and 2.44 μM, respectively. Their activities were comparable with amphotericin B (IC_50_ = 0.26 μM) and higher than sodium antimony gluconate (IC_50_ = 170 μM), miltefosine (IC_50_ = 13.60 μM), and paromomycin (IC_50_ = 8 μM). Compound **77** was evaluated for *in vivo* efficacy experiments and mechanistic assays. The ferrocenyl quinoline demonstrated curative properties for mice under *L. donovani* infection with a dose-dependent response. The *in vivo* therapeutic effect was achieved either upon noninvasive oral or invasive intramuscular administration. Then, several experiments focused on immunostimulation were performed for compound **77**. The water-soluble ferrocenyl quinoline **77** stimulated the secretion of Th1 either under oral or intramuscular administration. Furthermore, a significant induction in the expressions of key pro-inflammatory cytokines, IL-6, IL-12, TNF-α, and IL-1β, was observed at the mRNA level in the treated groups, either oral or intramuscular, in comparison to the infected group. By contrast, the mRNA expressions of the anti-inflammatory cytokines like IL-10 and TGF β were reduced for the *in vivo* model. Additionally, a significant increase in the level of NO in *vivo* models was found. The expression of pro-inflammatory cytokines, secretion of Th1, and reduction of expression of anti-inflammatory cytokines suggest that this type of compound must be inducing an immunostimulating response in the infected host. Further mechanistic experiments showed that compound **77** reduced the expression of some enzymes, such as γ-glutamylcysteine synthetase, glutathione synthetase, ornithine decarboxylase, and trypanothione reductase. To verify the induction of apoptosis, downregulation in the expression of Sir2 and enzymes NAD + biosynthetic pathway enzymes, nicotinamidase, Na phosphoribosyltransferase (NAPRT), NAMN adenyltransferase (NAMNAT), and ammonia-dependent NAD + synthetase were found under treatment of compound **77** against *in vitro* or *in vivo* model when compared with the untreated models. Finally, from the ADME-properties point of view, compound **77** showed a good pharmacokinetic and pharmacodynamics profile. In summary, compound **77** showed an effective leishmanicidal effect against *in vitro* and *in vivo* models of LV with curative properties, convenient ADME properties, and an immunostimulating response.

**FIGURE 23:**
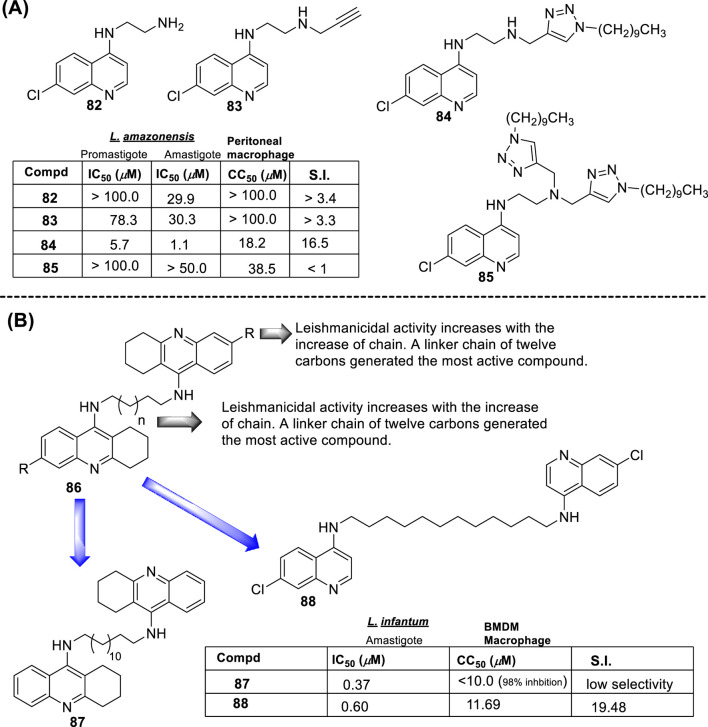
**(A)** Structure of 4-aminoquinolines with triazole rings at 4-diamine chain **82–85** reported by Glanzmann et al. (2021). **(B)** Structure of a quinoline dimer **86–88** with promising antileishmanial properties against *Leishmania infantum* promastigotes reported by Silva et al. (2023).

In 2021, Glanzmann and co-workers prepared a novel series of quinoline-triazole hybrids **82–84** (Figure 23A), and they were evaluated against *L. amazonensis* promastigotes and intracellular amastigotes (Glanzmann et al., 2021). Considering both promastigotes and amastigotes, the results demonstrated that only derivative **84** displayed a promising leishmanicidal response, with IC_50_ values of 5.7 µM and 1.1 µM against promastigote and amastigote strains, respectively. That compound was the most selective, displaying an SI value of 16.5, which was higher than typical 1 or 3 of the remaining compounds. On the other hand, mechanistic experiments based on compound **84** showed that it induces a significant reduction of the mitochondrial membrane potential, leading to the disruption of its function. Furthermore, a pronounced generation of ROS was noted under treatment with compound **84**. Apoptotic evidence was observed through cytometry. In conclusion, the introduction of a triazole ring induces an improvement in leishmanicidal response, leading to a specific mode of action through interference in the bioenergetic system and plasma membrane permeabilization, with subsequent activation of apoptosis-like and necrosis processes, culminating in cell death.

Finally, Tavares and co-workers developed a series of 1,2,3,4-tetrahydroacridines **86** based on a virtual screening performed against the enzyme S-adenosylmethionine decarboxylase and evaluated them against *L. infantum* promastigotes (Figure 23B) (Silva et al., 2023). Forty-two compounds were assayed. A SAR study was structured based on their results, and it was possible to verify that the length of the alkyl chain had a significant effect on the leishmanicidal activity of the compound, especially in the case of dimers. Most of the 1,2,3,4-tetrahydroacridines presented high levels of toxicity, with a percentage of macrophage death of about 90% at 10 µM. From the SAR analysis, only compound 87 was a promising candidate with an IC_50_ value of 0.37 µM, although high toxicity derived from an inhibition percentage of 98% in macrophage proliferation at 10 µM. Based on the relevance of a long alkyl chain as a linker between the quinoline core and the high toxicity of the acridine core, this research group decided to replace the tetrahydroacridine scaffold with a 7-chloroquinoline core to prepare compound **88**, which consists of two 7-chloroquinolines connected through an *N,N*-decan-1,10-diamine chain. The new compound showed a good leishmanicidal response with an IC_50_ value of 0.60 µM and a CC_50_ value of 11.69 µM, which implies a good SI value near 20. This replacement allowed the retention of the leishmanicidal response with decreased toxicity.

In summary, the following remarks about generating potent and selective compounds from the different 4-aminoquinolines can be extracted from these reports:

i) The inclusion of a chlorine atom at the 7-position has a detrimental influence;
ii) The incorporation of tertiary amine at the end of the alkyl-diamine chain at the 4-position is essential;
iii) The incorporation of lipophilic moieties like aryl- or extended alkyl chains either on the diamine chain or on the quinoline core improved potency and selectivity;
iv) An extensive alkyl-diamine chain can compromise the selectivity and increase toxicity;
v) A higher specificity toward the amastigote than toward the promastigote stage was found for most of the analyzed 4-aminoquinolines;
vi) The 4-aminoquinolines target the reduction of mitochondrial membrane potential, which promotes the reduction of ATP synthesis and ROS production that induces morphological alterations and death via apoptosis.

Table 1 summarizes the most highlighted compounds based on *in vitro* evaluation against intracellular amastigotes, cytotoxicity on macrophages, and a selectivity index higher than 10. From Table 1, compounds **16** (SI > 1111), **45** (SI = 145 vs. *L. amazonensis* and 392 vs. *L. infantum*), **54b** (SI = 41.3), **57** (SI > 250), **61** (SI = 41.6), and **76** (SI = 1739) are highlighted as the most promising candidate for further assays and as lead structures to inspire new design. These compounds are within the strictest parameters for the drug discovery of leishmanicidal agents (Don and Ioset, 2014). From the structural point of view, the most selective compounds (**16**, **45**, **57**, and **76**) are better adjusted to the strict parameters for the design of leishmanicidal agents (Don and Ioset, 2014). The inclusion of a lipophilic moiety (aryl or cyclohexyl) at the end of the 4-diamine chain, as well as the incorporation of a composed *N*-aniline moiety, such as 4′-phenoxy-aniline, into the structure of compounds **57** and **76** at the 4-position of the quinoline core is highly convenient for generating selective compounds as a consequence of the decrease of the cytotoxicity, conserving the leishmanicidal response. In particular, a comparison of compounds **57** and **76**, which bear an *N*-anilino moiety at the 4-position of quinoline core, showed that the incorporation of an extra basic moiety like phenyl-piperazine into compound **76** significantly enhances the selectivity compared with compound **57**. A comparison between 4-aminoquinolines featuring an alkyl-amine chain at the 4-position revealed that the inclusion either of a basic moiety or of a lipophilic moiety at the end of the amine chain improves the activity and selectivity, such as can be seen in compounds **16** and **45**. These features are also of great relevance for the accumulation in the host lysosome (Trapp et al., 2008) and parasite mitochondria (Duenas-Romero et al., 2007; Coimbra et al., 2010) as well as for promoting a consistent immunostimulant response acting as a TLR agonist/antagonist (Talukdar et al., 2021). Interestingly, when the size of the alkyl-amine chain at the 4-position is increased, the selectivity is compromised as a consequence of an increase of the cytotoxicity on macrophages, as can be seen in compounds **67** (SI = 8.2), **72** (SI = 13.1), **84** (SI = 16.5) and **88** (SI = 19.5). That tendency was appreciated in the quinolinic compound prepared by Bertinaria´s group (Guglielmo et al., 2009) (Figure 10), B.A. Solaja´s group (Konstantinović et al., 2018) (Figure 22), and Manzano et al. (2019) (Figure 21A). On the other hand, the ferrocenyl quinoline also proved to be a convenient scaffold for generating potent and active compounds against *in vitro* and *in vivo* models of VL. In addition, it is important to mention that the presence of an extra basic moiety is highly convenient for generating water-soluble compounds by the generation of protonated salts, such as can be seen for compounds **54b** and **78**, which is a physicochemical property that is highly desired for *in vivo* models using oral administration.

**TABLE 1:** Summary of promising leishmanicidal 4-aminoquinolines based on *in vitro* data.

Entry	Compound	IC_50_ amastigotes	CC_50_ macrophage	SI	*In vivo*	Reference
1	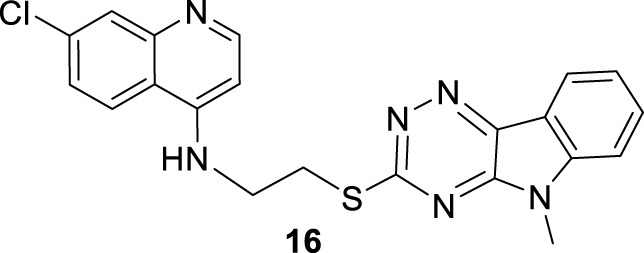	0.36 (*L. donovani*)	>400 (J774.1)	>1111	**NO**	Sharma et al. (2014)
2	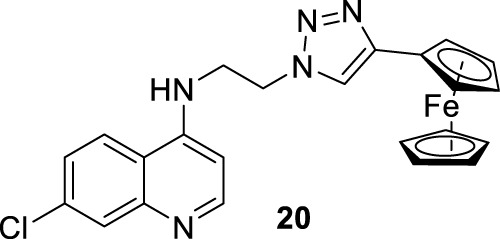	0.50 (*L. donovani*)	----	----	**YES**, Significant reduction of parasitemia	Yousuf et al. (2016)
3	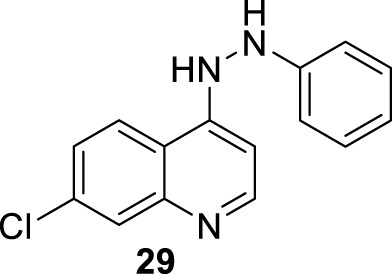	0.80 (*L. amazonensis*)	>30 (Murine peritoneal)	>38	**NO**	Antinarelli et al. (2015)
4	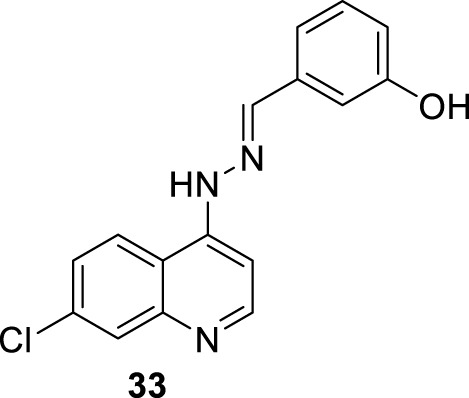	8.10 (*L. amazonensis*)	>150 (Murine peritoneal)	>18	**YES**, 94% reduction of parasitemia	Antinarelli et al., 2016; Antinarelli et al., 2020)
5	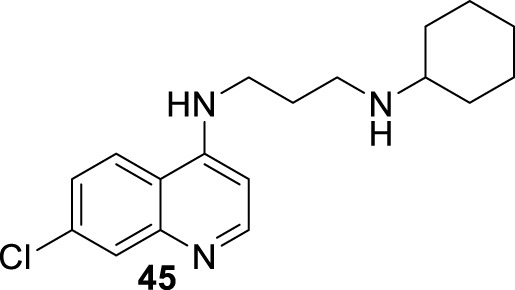	0.34 (*L. amazonensis*) 0.18 (*L. infantum*)	148.94 (Murine peritoneal)	145 (*L.a*) 392 (*L.i*)	**NO**	Sousa et al. (2019)
6	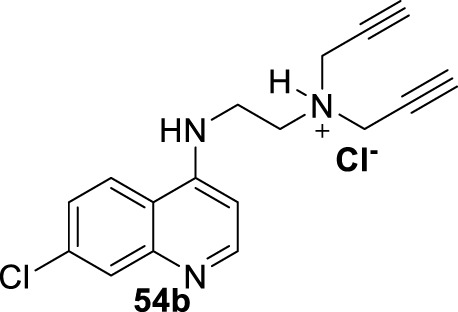	5.48 (*L. amazonensis*)	226.7 (Murine peritoneal)	41.3	**NO**	Calixto et al. (2018)
7	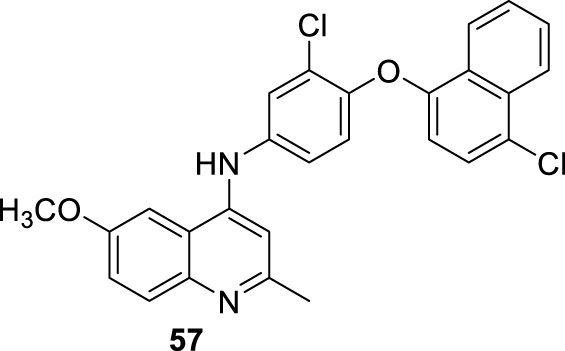	0.20 (*L. infantum*)	> 50 (J774.1)	>250	**NO**	Stevanovic et al. (2018)
8	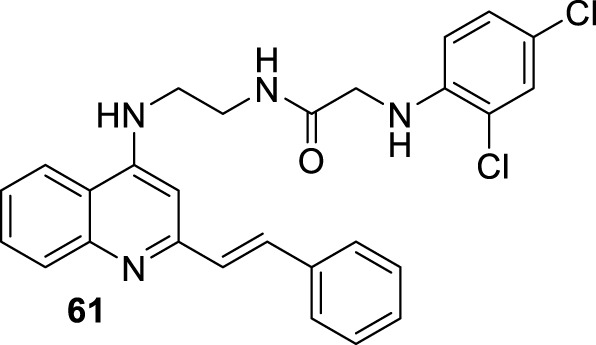	1.20 (*L. pifanoi*)	>50 (J774.1)	41.6	**NO**	Staderini et al. (2019)
9	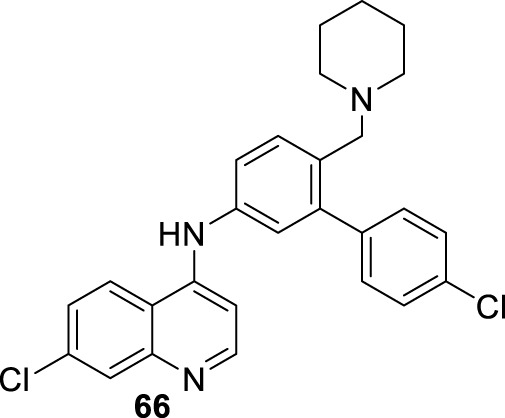	13.88 (*L. braziliensis*)	197.93 (Primary human fibroblast)	14.3	**NO**	Romero et al. (2019)
10	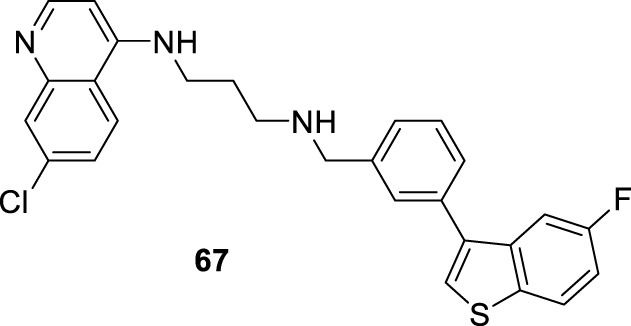	0.58 (*L. infantum*)	4.73 (THP-1)	8.2	**NO**	Manzano et al. (2019)
11	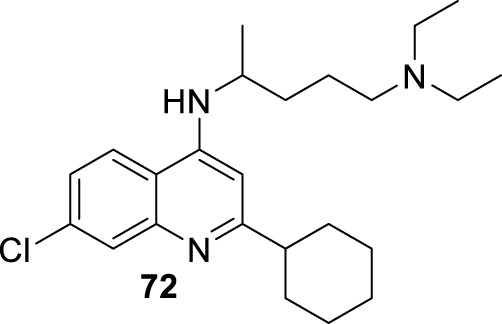	1.07 (*L. panamensis*)	14.03 (Murine peritoneal)	13.1	**YES**, reduction of parasitemia, lesion size, and inflammation	Herrera et al. (2016)
12	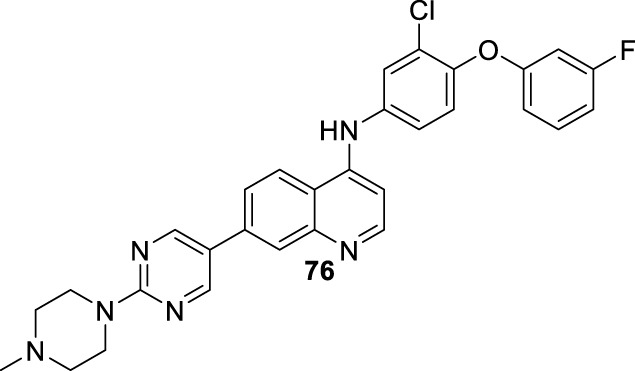	0.023 (*L. donovani*)	40 (HepG2)	1739	N**O**	Singh et al. (2020)
13	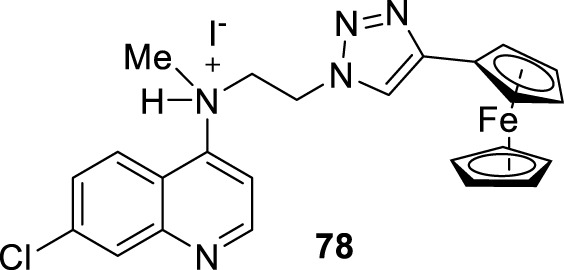	0.50 (*L. donovani*)	----	----	**YES**, curative properties, reduction of parasite load, and reduction of IL-10 and TGF β	Mukherjee et al. (2020)
14	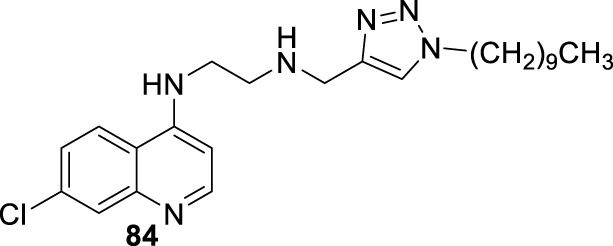	1.10 (*L. amazonensis*)	18.2 (Murine peritoneal)	16.5	N**O**	Glanzmann et al. (2021)
15	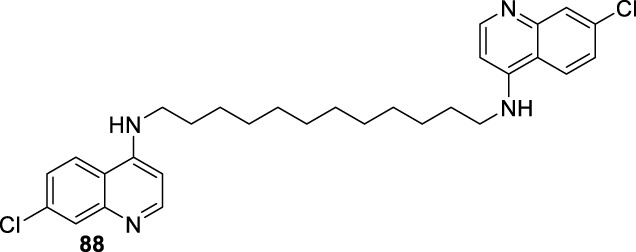	0.60 (*L. infantum*)	11.69 (BMDM)	19.48	**NO**	Silva et al. (2023)

A few of the compounds mentioned in Table 1 have been proved against *in vivo* models of VL. In particular, compound **45** was proved against an *in vivo* model of VL of *L. infantum*, promoting a significant reduction of parasitemia (66%–72%) larger than that promoted by amphotericin (42%–62%). Other groups of compounds, including compounds **20**, **33** (SI > 18), **72** (SI = 13.1), and **78**, displayed a good *in vivo* efficacy with parasitemia reduction. In particular, for compounds **20** and **78**, beyond the *in vivo* efficacy, an immunological activation was proved through the increase of pro-inflammatory cytokines. Compound **78** is an attractive alternative to favor the administration and pharmacokinetics due to its solubility in water.

From the synthetic point of view and based on promising compounds shown in Table 1, compounds **20, 29, 33, 45, 54b, 57, 78, 84**, and **88** can be prepared from simple and short synthetic routes involving a simple or three-step simple route, requiring only the extra functionalization of 4-alkyl amine substitution and the final step of nucleophilic aromatic substitution at the 4-position in 4,7-dichloroquinoline substrate. Meanwhile, the synthesis of compounds **16, 61, 67, 72**, and **76** require complex synthetic routes involving several steps as a consequence of specific functionalization at the quinoline core as well as the incorporation of complex functionalization at the 4-amine chain. In summary, compounds **45** and **78** are attractive scaffolds due to their significant leishmanicidal activity against *in vitro* and *in vivo* models of VL and also due to their synthetic feasibility and physicochemical properties for oral administration. Meanwhile, compounds **16** and **76** emerge as promising candidates for further studies to evaluate their potential as leishmanicidal agents.

## 4 Conclusion

In the last decades, compounds based on 4-aminoquinoline have shown great potential for the drug discovery of leishmanicidal agents with a well-defined mechanism of action. The present review showed how the 4-aminoquinolines possess a high specificity toward the amastigote strain over the promastigote, which is highly relevant because the first is the stage in clinical manifestations. Furthermore, a SAR analysis comparison based on diverse examples showed that two factors, basicity and lipophilicity, are key parameters leading to a significant leishmanicidal response and are selective. In particular, a tertiary terminal amine and long hydrocarbon chains are required. From the mechanistic point of view, it was found that 4-aminoquinoline targets two key organelles, the lysosome macrophage and parasite mitochondria as well as playing a role in immunostimulation. The incorporation of lipophilic chains is essential when targeting macrophage lysosomes, and fundamental reports indicate that Log P values between 4 and 6 are required. Once within the lysosome, the 4-aminoquinoline enters the parasite, and under the protonated form (under an acidic lysosome pH), the compounds target the mitochondria, where they promote depolarization of mitochondrial potential, which is key to conserving energy production. This latter favors ROS production, decreases ATP production, and promotes parasite apoptosis. Additionally, 4-aminoquinolines could act as TLR agonists or antagonists, which could favor macrophage activation and activate the innate immune macrophage system, which emerges as an extra defense mechanism to attack parasites within the macrophage. Interestingly, a more selective TLR response is found for 4-aminoquinolines with terminal tertiary amine and lipophilic groups.

The present review provides a considerable update on development, taking into account two key aspects: (i) SAR analysis and (ii) identification of key targets and their modulation based on SAR analysis. The information seeks to give the scientific community a more in-depth understanding of the most suitable functionalizations to perform in this particular family of compounds for the construction of potent and selective leishmanicidal agents and how it is possible to modulate the mentioned targets. Our report is a starting point to inspire future investigations directed to the design of leishmanicidal agents based on 4-aminoquinolines.
